# Healthcare worker practices for HPV vaccine recommendation: A systematic review and meta-analysis

**DOI:** 10.1080/21645515.2024.2402122

**Published:** 2024-10-14

**Authors:** Damola Bakare, Elisa Gobbo, Kofoworola O. Akinsola, Ayobami A. Bakare, Julius Salako, Claudia Hanson, Sibylle Herzig van Wees, Adegoke Falade, Carina King

**Affiliations:** a Department of Paediatrics, University of Ibadan, Ibadan, Nigeria; b Department of Global Public Health, Karolinska Institute, Stockholm, Sweden; c Department of Paediatrics, University College Hospital, Ibadan, Nigeria; d Department of Community Medicine, University College Hospital, Ibadan, Nigeria; e Department of Disease Control, London School of Hygiene and Tropical Medicine, London, UK; f Centre of Excellence for Women and Child Health, Aga Khan University, Nairobi, Kenya

**Keywords:** Human papillomavirus, healthcare workers, vaccine confidence, recommendation behavior, systematic review, meta-analysis

## Abstract

Healthcare workers (HCWs) are trusted sources of information for vaccination and their attitude toward vaccination is thus critical. We aimed to synthesize existing literature on healthcare workers’ HPV vaccine confidence and their practices of recommending this vaccine. We conducted a systematic literature review and meta-analysis, with the search conducted last in March 2024. For the inclusion criteria, the studies needed to include healthcare worker practices or behaviors on recommending the HPV vaccination. Seventy-three articles were included. The proportions of HCWs recommending varied considerably by region and gender of the recipient, but there was no statistically significant difference in income level or pre- or post-HPV vaccine introduction into the national vaccination program. The main barriers to recommending HPV vaccination were concerns around safety and efficacy, cost, parental concerns, and systemic barriers. The results illustrate the importance of contextually adapted approaches to improving vaccine acceptance and recommendation.

## Introduction

Human papilloma virus (HPV)-related cancers affect approximately 625,600 women and 69,400 men per year, with cervical cancer being the most common HPV-related cancer.^1^ Despite being preventable, cervical cancer remains one of the leading causes of cancer-related deaths among women worldwide.^2,3^ In 2020, the global incidence rate of cervical cancer reached 13.3 cases and resulted in 7.2 deaths per 100,000 women-years^4^ with significantly higher incidence and mortality rates in low- and middle-income countries (LMICs).^5,6^ The World Health Organization (WHO) advocates two primary prevention strategies for cervical cancer: the widespread adoption of the HPV vaccine and cervical cancer screening programs.^7^

Although the HPV vaccine has demonstrated efficacy in protection against major HPV strains associated with cervical cancer,^2,8,9^ global vaccination coverage remains low.^10^ In 2021, an estimated 12.2% vaccine coverage was reported amongst 15-year-old females globally, ranging from 51.5% coverage in high-income countries to 11.3% in low-middle-income countries.^10^ This low coverage has been attributed to the limited availability of the vaccine given its high cost in some settings, but also due to safety concerns about the vaccine among adolescents, parents, and communities.^11^

MacDonald et al. define vaccine hesitancy as a “*delay in acceptance or refusal of vaccines, despite vaccine available services*” and is a complex and context-specific phenomenon.^12^ Vaccine hesitancy has been defined as both a behavior and a state of indecisiveness, which is characterized by the WHO 3C model: Confidence, Complacency, and Convenience.^12,13^ Confidence refers to trust in the vaccine’s safety, efficacy, and the system delivering the vaccine, healthcare workers (HCWs), and policy makers.^a^ Complacency denotes the perception of a low risk of disease and the belief that vaccination is unnecessary, while convenience involves factors such as vaccine availability, affordability, and willingness-to-pay for vaccines.^12^ Understanding these dimensions for different contexts and vaccines is fundamental to increasing coverage.

Healthcare workers (HCWs) are considered trusted sources of information about vaccines,^14,15^ play a vital role in shaping public perceptions, and are influential in improving vaccine uptake.^15–17^ However, globally, declining vaccine confidence among HCWs, is a major public health concern.^17,18^ Previous reviews have examined HCWs perceptions and knowledge on vaccines^14^ and their recommendation quality for adolescents in the USA.^19^ To expand on this literature, we aimed to review existing literature measuring HCW practices on recommending the HPV vaccine globally. By synthesizing and critically evaluating available studies, we aim to elucidate the factors influencing HCW recommendations for the HPV vaccine, providing insights for global efforts aimed at enhancing vaccine confidence and uptake.

## Materials and methods

We conducted a systematic literature review based on the following research question: What are health care workers’ recommendation behaviors for the human papilloma virus (HPV) vaccine? The full systematic review protocol is available in Appendix 1.

### Search strategy

A literature search was performed in June 2023 and re-run in March 2024, in the following databases: Medline, Web of Science, CABI: CAB Abstracts, and Global Health and Sociological Abstracts and as a complementary search Publicly Available Content database was used. The search strategy was developed in Medline (Ovid) in collaboration with librarians at the Karolinska Institutet University Library. For each search concept Medical Subject Headings (MeSH-terms) and free-text terms were identified. The search was then translated, in part using Polyglot Search Translator,^20^ into the other databases. The strategies were peer reviewed by another librarian prior to execution.

Key search terms used were (immunization, immunization programs, exp vaccination, exp vaccines) and (anxiety, awareness, behavior, choice behavior, communication barriers, health knowledge, attitude, and practice, intention) and (health personnel, benchmarking, health care surveys, quality assurance, health care, survey and questionnaire). A de-duplication process was done using the method described by Bramer et al. (2016) and DOIs were compared to avoid duplicate articles.^21^ The full search strategy is available in Appendix 2.

### Eligibility criteria

We included original peer-reviewed articles that focused on vaccine hesitancy/confidence, behavior, or attitudes, with data on HPV vaccine recommendation behavior or practices. The population group studied were HCWs, which includes physicians, nurses, pharmacists, and healthcare administrators.^22^ Dentists and students were excluded since they are not directly involved in vaccination. There were no language restrictions applied, and the databases were searched from inception.

### Selection process

We used Rayyan.ai^23^ software for the screening process which also allowed for blinding. In the first round of screening, EG created a shortlist including any articles measuring HCW vaccine confidence/hesitancy/acceptance. The articles from this shortlist were rescreened in February 2024 to double check for any potentially missed articles. Then, two researchers (EG and KA) did a blinded title and abstract, and then full-text screening. Any discrepancies in screening between the researchers were discussed with the wider team for final inclusion. More information is provided in Appendix 1.

### Data extraction

We employed a two-tiered approach for data extraction and analysis. First, we collected descriptive data from each study, including publication year, study design (e.g., cross-sectional, mixed methods, or qualitative), sample size, participant demographics (such as type of healthcare worker), and geographical location (country and WHO region). The data was divided so that two researchers (KA and EG) extracted data and conducted the quality assessment on two-thirds of the articles, and another two researchers (DB and JS) did the remaining third. This allowed for all the articles to be extracted and screened by two researchers. The entire process was blinded among all the researchers until after extraction and quality assessment was completed on all articles.

Data was extracted using a form based on the research question and guidance from the JBI systematic review framework.^24^ For the meta-analysis, we extracted data on the study country and used World Bank income-level definitions to categorize by income, if the publication was before or after the official inclusion of the HPV vaccine in the study setting’s national guidelines, and data on total sample size, total recommending, and total willingness to recommend (including sub-groups for boys and girls). We accounted for variation in measurement tools by including any item on HCW recommendation (should, would, or do) and distinguishing between actual recommendation behaviors (practical application) and those assessing willingness to recommend (intent or inclination). Where data differentiated recommendation practices or willingness by gender or age, we further separated these into distinct sub-analyses.

### Quality assessment

The quality assessment tools used were the JBI cross-sectional study^25^ and JBI qualitative study,^26^ depending on the study design. No articles were excluded on quality concerns only. However, we did take note of articles that conducted some form of validation or reliability check on the tool utilized, and that adjusted appropriately for confounders.

### Data analysis

We first described key study characteristics using means and proportions and conducted a narrative synthesis of the principal findings concerning HCW attitudes and behaviors related to recommending the HPV vaccine. This step involved summarizing the main outcomes, identifying reported barriers and facilitators to vaccine recommendation and examining any additional relevant information.

We then performed a meta-analysis including all studies that reported quantitative measures of either past recommendation practices or current willingness to recommend the HPV vaccine. These papers used a variety of measures. We defined recommending behavior and willingness to recommend as binary variables. Questions about how often (i.e. always/never) or how strongly HCWs recommend the HPV vaccine were used to assess recommending behaviors, while willingness to recommend was defined as an intent or interest in recommending HPV. We used binary measures/responses to obtain the proportion of HCWs’ recommendation behavior or willingness when a binary measure was used directly. If the authors generated a binary outcome from a scale (i.e., Likert scale) we used that, and if there no binary measure was provided in the article, we derived a binary from the top two to three responses (i.e. Always, Almost Always, Sometimes or Strongly Agree, Agree, Neutral).

To accommodate potential variability among the studies, we applied random-effects meta-analyses, using the metaprop command in Stata SE18,^27^ which estimates a pooled proportion and exact binomial confidence intervals. When studies reported multiple age groups, we used the mean proportion for our calculations. We also conducted sub-group analyses by study design (limited to cross-sectional and mixed methods), the income status of the study setting (according to the World Bank classification), WHO region, and the publication date in relation to when the HPV vaccine was officially included in the study countries national guidelines. These sub-groups were selected due to their potential influence on HPV vaccine uptake and coverage, and completeness of reporting.

## Results

**Figure 1.**
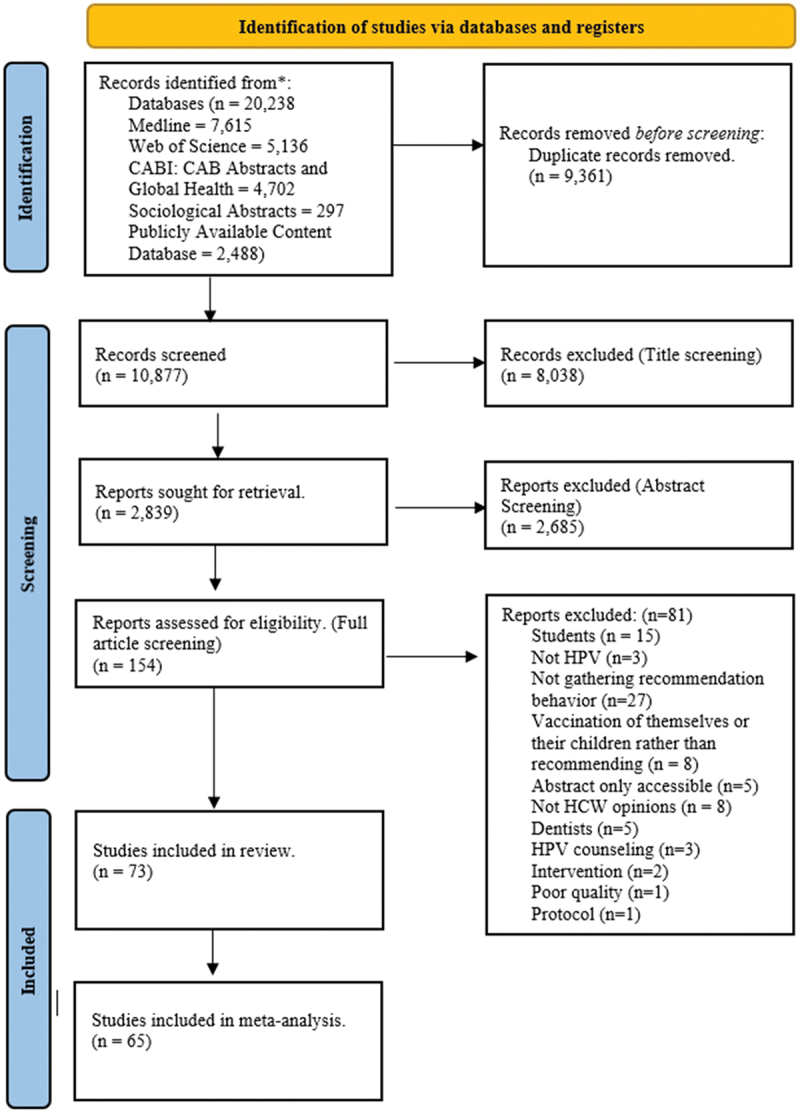
PRISMA flow diagram for systematic review article inclusion.

**Figure 2.**
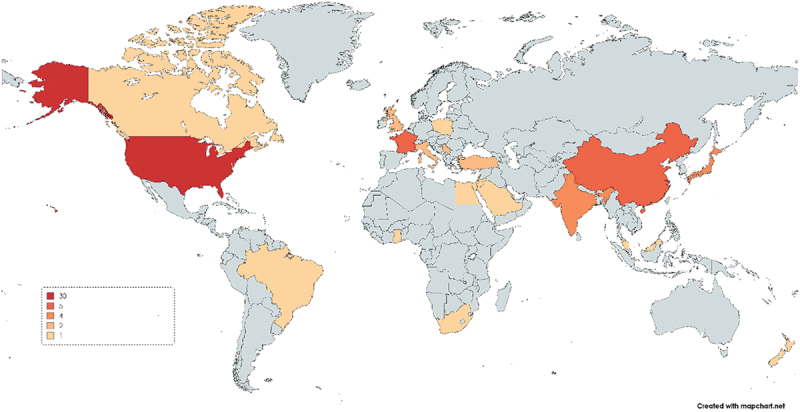
Global map with number of articles per country (MapChart)*.

Overall 10,877 articles were returned from the search, 73 articles were selected for final inclusion, and 65 were included in the meta-analysis (Figure 1). Sixty-five of the articles used a cross-sectional design,^28–91^ four used mixed-methods,^44,92–94^ and four had a qualitative study design.^95–98^ All the articles were published since 2005, which is 1 year prior to when the HPV vaccine first became available in the USA.^99^ The sample sizes in the cross-sectional studies varied widely with a mean of 525 and median of 245 participants (range: 9–5270). The vast majority of studies were conducted in high-income countries with 73% (53/73) articles in high-income countries,^29–31,34,35,37–39,41,43–51,54–67,69,70,72–74,79,81,83,85–94,97,98,100^ 11 in upper-middle,^33,36,40,53,71,75–78,82,84^ 8 in lower-middle,^28,32,42,52,68,80,95,96^ and none in a low-income country, as illustrated in Figure 2. Notably, 41% (30/73) of the studies were done in the USA, which is where the HPV vaccine was first introduced in the world.^30,31,35,38,39,41,43–45,49,55,56,62,63,65,67,70,74,79,81,83,86,88–91,94,97, 100^ A full summary of the included 73 articles is presented in Appendix 3.

*One article^80^ had participants from 23 African countries, but since the specific countries were not named by the authors this article was not included in this map.

### Meta-analyses of recommendation behavior

**Figure 3.**
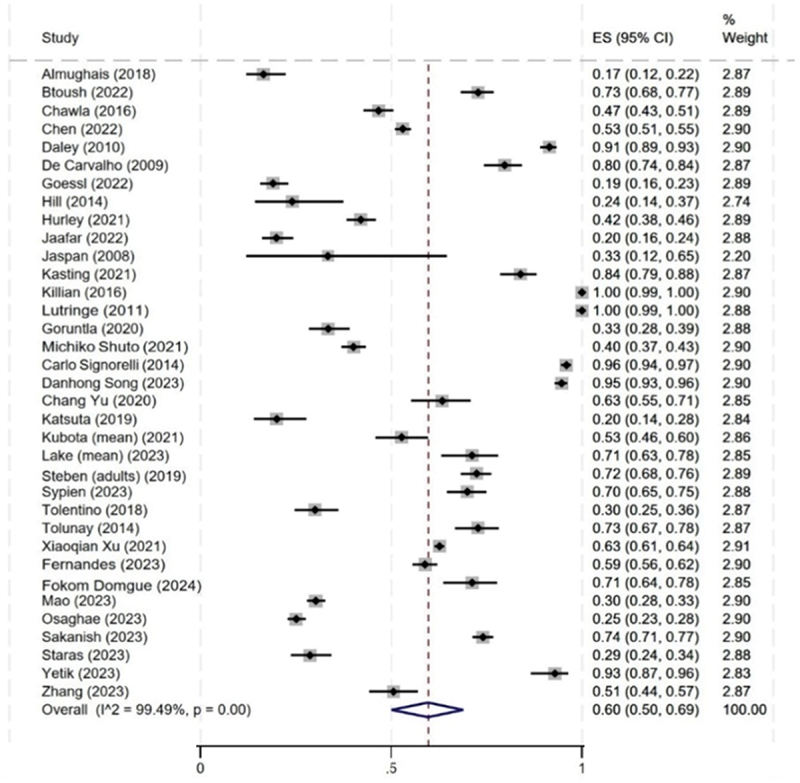
Proportion of healthcare workers recommendation behavior (practice) for both boys and girls (*n* = 35).

From the meta-analysis results, we observed substantial heterogeneity in the proportion of HCW who reported previously recommending the HPV vaccine (or expressing a strong recommendation) across the included studies. Figure 3 presents the overall Forest plot for HCWs’ HPV recommendation behavior, with 35 articles presenting this data; the pooled proportion was 60% (95% CI: 0.50, 0.69). However, the proportion varied considerably, ranging from nearly 100%,^52,92^ to less than 20% of HCWs recommending the HPV vaccine.^29,38^ The use of different measures and target populations may have contributed to this variability along with different levels of hesitancy.

**Figure 4.**
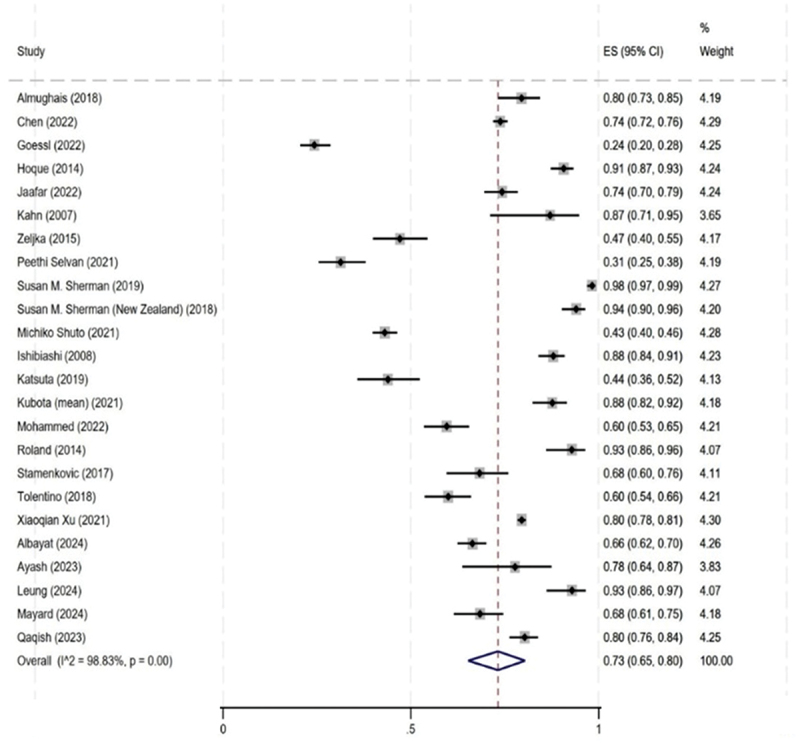
Proportion of healthcare workers recommendation willingness (intent) for both boys and girls (*n* = 24).

We found a higher overall proportion of HCWs expressing willingness to recommend the HPV vaccine (0.73 [95% CI: 0.65, 0.80]) compared to those who reported having already recommended it (0.60 [95% CI: 0.50, 0.69]). Twenty-four studies assessed HCWs’ willingness (or intent) to recommend the vaccine, and the findings are presented in Figure 4. Across these studies, more HCWs cited a willingness to recommend than those who strongly recommended in practice.^29,33,40,42,53,68–71,76,78,84,100^

**Figure 5.**
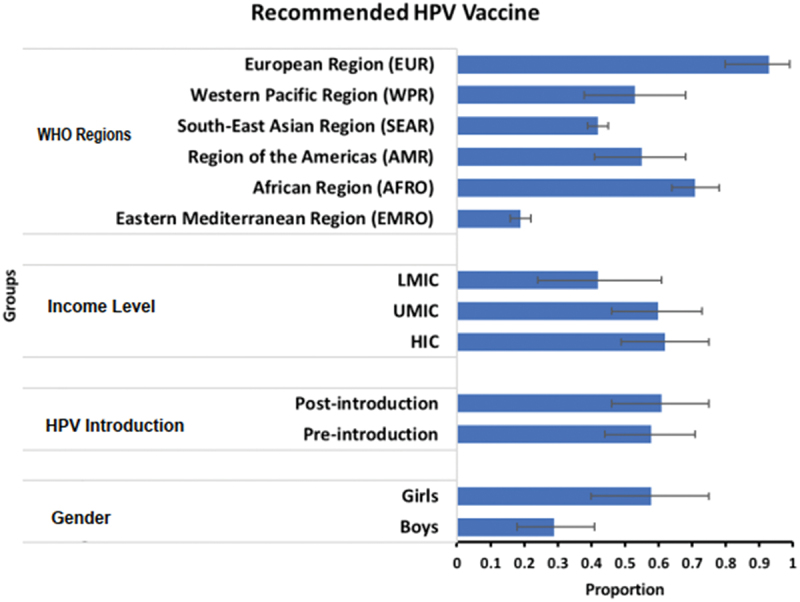
Bar chart of overall proportion for the sub-analyses of WHO region, income level, HPV introduction time, and gender.

The sub-group analyses illustrate some of the factors behind this wide variation in results – Figure 5. Ninety-three percent (95% CI: 0.80, 0.99) of HCWs from the European region reported that they are recommending the HPV vaccine, whereas in the Eastern Mediterranean region only around 19% (95% CI: 0.16, 0.22) are recommending the HPV vaccine. Appendix 4 illustrates willingness to recommend by subgroups (region, income level, and HPV introduction). There was no significant difference in recommendation behavior by income status (low income = 0.42, upper middle income = 0.60 and high income = 0.62) or pre-post introduction (pre-introduction = 0.58 and post-introduction = 0.61). Meanwhile, there was a greater proportion of HCWs who have recommended to girls compared with boys (in studies where this was assessed separately), 58% (95% CI: 0.40, 0.75) and 29% (95% CI: 0.18, 0.41), respectively (Appendix 5).

### Facilitators and barriers

**Figure 6.**
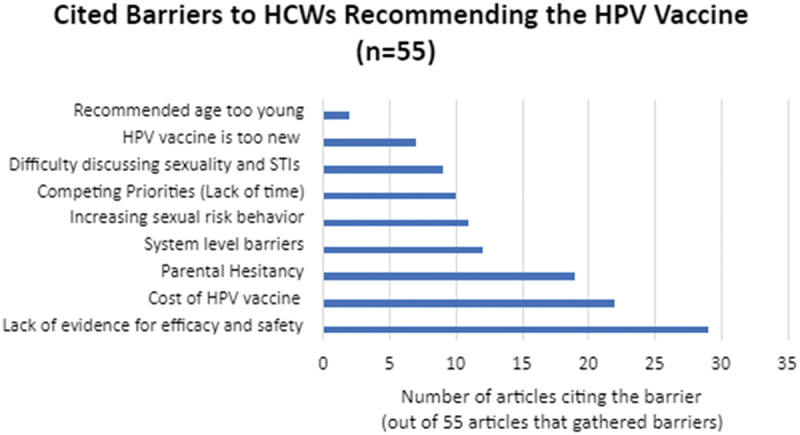
Cited barriers to recommending HPV (*n*=55).

To understand what influences HCWs’ recommendation behavior, we analyzed the studies for reported facilitators and barriers to HCWs confidence in recommending the HPV vaccine. Fifty-five of the studies gathered information specifically on barriers as part of the study.^28,30,31,33–37,39,42–45,47–59,61–64,66,69–78,80,82–84,86,91–98,100,101^ Figure 6 illustrates the main barriers cited by the HCWs in the included studies. The most cited barriers to recommending were concerns about the efficacy or safety of the vaccine,^31,33,34,36,42,44,45,51–54,56,57,59–61,63,64,66,69,73,75,78,82,84,91,93,95,96^ the cost of the HPV vaccine,^28,34,35,39,42,43,45,49,52,53,55,56,61–63,69,71–73,75,80,94,96,97^ and concerns about parental vaccine hesitancy.^28,35,43,44,47,49,51,55,60,69–72,74,76,82,91–94,96–98,100^

Facilitators were not explicitly gathered by the articles, but several presented some demographic characteristics associated with HCWs who were willing to recommend the HPV vaccine. Among the relevant studies shown in Appendix 6, 19/21 found female providers versus male providers,^28,29,31,33–35,48,53,69,72–75,80,82,86,88,89,95^ 17/17 found those with higher knowledge scores,^28,29,31,45,46,48,61,73,74,78,79,82,84,87,89,95,100^ 10/13 found younger providers versus older,^29,33,44,52,68,69,86,88,92,100^ 8/9 found OB/GYNs versus other physician groups,^42,54,64,73,75,78,82,89^ and 4/4 found urban providers versus rural were more likely recommend.^38,51,77,86^

### Quality of included studies

Thirty-nine out of 65 (60%) of the studies that used a cross-sectional design reported a form of psychometric validity or reliability testing of the survey tool used.^29,31,34,35,37,45–47,49–52,56,57,59,61,62,64,65,67–76,78–80,84,86,88,91,94,100^ These studies either conducted some form of validation testing on the survey questions used or used a survey tool that has previously been validated to measure vaccine hesitancy and provider recommendation behavior. One of the limitations is that there is not a widely accepted or common survey tool that is validated to assess provider willingness to recommend vaccines.^102^

Another important quality assessment is to determine if the studies adjusted for confounders. Out of the 65 cross-sectional studies, 23 studies^32,33,39,41,43,50,52,54–57,59,60,65,72–75,77,84,86,94,103^ did not fully control for confounders, based on the research team’s quality assessments (available in Appendix 7). For the qualitative and mixed-methods studies, they generally fulfilled the quality assessment criteria, but only one study revealed the role of researcher within the study.^98^

## Discussion

We aimed to review existing literature exploring HCW behavior toward recommending the HPV vaccine. 73 articles that measured HCW attitudes toward recommending the HPV vaccine were included in the review. We found significant differences in HCW recommendation practice across the WHO regions from over 90% in the European region to less than 30% in the EMRO. There was also a statistically significant difference in HCWs recommending to girls compared with boys. While other the factors of income and HPV vaccine introduction did not demonstrate any statistical significance. Additionally, there are more HCWs that indicated a willingness to recommend the HPV vaccine compared to HCWs who have previously recommended it. This illustrates a gap in HCW workers’ intention and actual behavior. Similarly, a 2021 systematic review of 17 vaccines including HPV vaccine, found that there is a significant prevalence of HCW vaccine hesitancy related to inadequate knowledge, low confidence, and low self-vaccination.^14^ It is critical to examine and address this hesitancy as HCWs are trusted information sources and can influence patient or parental vaccine decision-making.^14,16^ These systematic reviews and meta-analysis results indicate mixed levels of HCW HPV vaccine confidence and that it varies by geographic location and gender of the vaccine recipient.

A deeper analysis shows the ways the 3Cs of confidence, complacency, and convenience impact HCWs’ recommendations. One notable barrier to HCW vaccine confidence was concerns around parental hesitancy. A report by the European Centre for Disease Control provides resources on strategies to address parental hesitancy or refusal.^104^ Inclusion of these strategies, such as a framework for communicating,^105^ motivational interviewing,^106^ and the SARAH (Strategies and resources to assist hesitant parents with vaccination) method,^107^ into HCW vaccine training could alleviate some of these concerns. This should focus on building confidence by addressing concerns about vaccine safety, efficacy, and side effects through trusted expert-led education and open communication.

Another confidence-related barrier was that HCWs were slightly more hesitant in settings where the HPV vaccine was not yet introduced into the official vaccine schedule. A few studies also mentioned the HPV vaccine being “too new” as a barrier to recommending the vaccine. Larson et al. report that there are noted population level increases in vaccine hesitancy surrounding new policies or newly reported vaccine risks, and they draw particular attention to hesitancy surrounding the MMR, COVID-19, and HPV vaccines.^65^ However, the difference in proportion was very small, and so it is important to consider the barriers to HCWs recommending during both the introduction of the HPV vaccine and even years later. Additionally, similar to the findings from Kong et al. in the United States,^19^ higher knowledge levels were associated with a higher likelihood to recommend. These illustrate the importance of ensuring good HCW vaccine education as a method for improving general population vaccine uptake.

There also appeared to be limiting factors around the convenience of recommending the vaccine due to competing interests. In several articles, the HCWs mentioned a lack of time as a limitation to recommending the HPV vaccine. This indicates that the HPV vaccine may not be of a high priority within their workflow due to logistical constraints. A few other studies have similarly shown that HCWs do not feel they have adequate time or resources to address vaccine hesitancy.^14,108,109^ This could be a possible explanation for the notable gap between intention to recommend and actual vaccination practice. Thus, streamlining vaccination access by offering on-site clinics, reducing administrative burdens associated with vaccination, being well trained in vaccine education, and having prepared information resources could improve convenience for HCWs.

The studies also revealed challenges relating to system-level concerns around cost, cold-chain management, and access to the HPV vaccine. In a qualitative study in Nigeria,^110^ a similar result was found that HCWs had lower confidence due to the precarious nature of the health system. Thus, our findings suggest a multifaceted approach is needed while addressing HCW vaccine hesitancy, similar to parental hesitancy.

Additionally, the group analyses of the meta-analysis revealed stark variation in vaccine confidence by region. Particularly, complacency and cultural barriers around HPV vaccination seems to be a concern in the EMRO region. In this region, there was a notably lower proportion of HCWs recommending the vaccine compared with the other WHO regions. Among the nine studies conducted in the EMRO region, six reported that HCWs cited cultural reasons or difficulty discussing sexuality and sexually transmitted diseases as a barrier to recommending.^28,42,68,69,75,84^ In another review on the impact of Islam on HPV vaccine acceptability in the Middle East and North Africa, Hamdi explains that countries in this region often have more traditional norms and, thus, it is thought that if the tenets of Islam regarding sexuality are followed then the risk of HPV or other sexually transmitted diseases (STDs) is very low.^111^ This contributes to a false belief, due to limited reporting, that STDs are rare in this region.^111^

These preconceived judgments that HPV and other STDs are not relevant make the HCWs become complacent in this region. Our results similarly illustrate that HCWs in the EMRO region felt that the HPV vaccine was not needed in their setting^68,69,84,85^ or mentioned other cultural or religious barriers to recommending the HPV vaccine.^28,42,68,69,75,84^ Especially, as the sociosexual behaviors begin to drastically change in the region (more youth participating in premarital sexual behaviors and getting married at older ages) and the subsequent increased risk of STD and HPV transmission in the region, there is an urgent need to have culturally relevant strategies to improve vaccine acceptance among HCWs.^111^

Additionally, Western Pacific and South-Eastern Asia Regions also both had slightly below half of HCWs recommending the HPV vaccine overall seemingly due to cultural confidence and complacency-related concerns. An article from Wong et al., explains that there are many social and cultural norms influencing HPV vaccine uptake in Asia, such as: parental concerns around the vaccine increasing sexual risk behavior, complacency due to religious beliefs to only engage in marital sexual activity, stigma of promiscuity associated around taking the vaccine, beliefs in local remedies, the vaccine being considered non-halal, and concerns around the rise of manufacturing from India and China.^112^ Similarly to the EMRO region, Wong et al., recommend that social-culturally sensitive approaches using community and religious leaders are needed to better overcome vaccine hesitancy in Asia.^112^

Contributing to the lack of confidence and trust in the vaccine and the institutions that prompt it, the Japanese government suspended the recommendation of the HPV vaccine between July 2013 and November 2021 due to highly publicized alleged adverse events, which sowed public distrust of the vaccine.^113–115^ Among the four articles from Japan in our review, none of them had more than 50% always/often recommending the HPV vaccine.^54,59,64,87^ Thus, in Japan, it is particularly important to disseminate accurate information about cervical cancer, HPV, and the HPV vaccine^14,104–110,114^

### Gaps in literature

Overall, we found strong evidence on HCW HPV vaccine hesitancy in some regions, but some gaps remain. The literature covers many different types of healthcare workers and patient groups but lacks socioeconomic diversity. Currently, there are very few studies (8/73) in LMICs. This could be because many LMICs have not yet or recently introduced the HPV vaccine into their routine immunization programs. Since 2019, 52 countries have introduced the HPV vaccine, many of which are LMICs,^116^ thus we would anticipate fewer studies in these countries.^21,26,36,46,59^ However, this could also be due to limited resources or disease burden prioritization in these areas^117^ hence further research should be done in LMICs particularly because we found an indication of high HCW HPV vaccine hesitancy, and because of the higher incidence rate of cervical cancer and lower vaccination uptake in those countries.^7^

### Limitations

There are some limitations to our study. First, due to the large number of studies found in the search, two sets of researchers conducted the extraction and quality assessment. This could have led to some variety in the results, but we ensured that at least two researchers extracted data and conducted a quality assessment on each article included in the review. Second, for the meta-analysis the articles did not have consistent tools or items that they measured, and thus the proportions were calculated from a diversity of measures.^118^

Lastly, the systematic review demonstrates that only slightly over half (39/67) of all the included studies utilizing a survey or questionnaire described a clear tool validation and reliability procedure. The studies were either not conducting validity testing or not using an existing validated tool, or not presenting it in the articles, which may diminish the trustworthiness of the results presented. Utilizing well-validated tools is critical for ensuring that the tools are reliable, accurate, and effective ways to measure HCW vaccine confidence and sentiments on recommendation.^118^ Thus, we recommend that future studies on HCW vaccine confidence attempt to use a standardized and validated survey tool.

## Conclusion

HCWs are critical for supporting vaccine acceptance and uptake as they are one of the most trusted sources of vaccine information.

It is valuable to use this existing research to help inform efforts to improve HCW HPV recommendation practice. Our results show that there are notable levels of HPV vaccine hesitancy among HCWs, but HCWs’ recommendation behaviors vary based on many factors, including geographic region of practice and concerns around parental hesitancy. This indicates a need for more contextual relevant approaches to addressing HCW vaccine hesitancy among HCWs. Additionally, efforts need to address HPV vaccine literacy around concerns of safety and efficacy about the HPV vaccine, and other systemic-level changes (cost, stocking issues, physical accessibility) are important to ensure HCWs are confident and able to recommend the HPV vaccine.

## Supplementary Material

Appendix 7_Quailty assessment of included articles.pdf

Appendix 6_Potential facilitators of HCW recommendation.docx

Appendix 3_Summary table of included articles.docx

Appendix 2_Documentation of search strategies.docx

Appendix 1_Healthcare worker and HPV systematic review protocol.docx

Appendix 4_Proportion of HCW recomendation willingness.docx

Appendix 5_Proportion of HCW recommendation behavior.docx

## Data Availability

The datasets used and/or analyzed during the current study are available from the corresponding author on reasonable request.

## Note

[a] Vaccine hesitancy, vaccine confidence, and vaccine acceptance are used interchangeably throughout the literature, but do have varying definitions and focuses. For the purpose of this paper, we will use the term vaccine confidence.
