# Supplementary material for: Healthcare worker practices for HPV vaccine recommendation: A systematic review and meta-analysis
Source: Hum Vaccin Immunother. 2024 Oct 14;20(1):2402122. doi: 10.1080/21645515.2024.2402122 (PMC11486212; doi:10.1080/21645515.2024.2402122)
Supplement: Appendix 7_Quailty assessment of included articles.pdf [file KHVI_A_2402122_SM6235.pdf]

	Abi Jaode, et al.	Almughais, et al.	Albayat, et al	Ayash, et al.	Ayres, et al.	Btoush, et al.	Chawla, et al.	Chen, et al	Daley, et al
1. Were the criteria for inclusion in the sample clearly defined?	Yes	Yes	Yes	Yes	Yes	Yes	Yes	Yes	Yes
2. Were the study subjects and the setting described in detail?	Yes	Yes	Yes	Yes	Yes	Yes	Yes	Yes	Yes
3. Was the exposure measured in a valid and reliable way?	Yes	Yes	Unclear	Not applicable	Yes	Yes	Yes	No	Yes
4. Were objective, standard criteria used for measurement of the condition?	Yes	Yes	Yes	Yes	Yes	Yes	Yes	Yes	Yes
5. Were confounding factors identified?	Yes	Yes	Yes	No	Yes	Yes	No	Yes	Yes
6. Were strategies to deal with confounding factors stated?	Yes	Yes	Yes	No	Yes	Yes	No	Yes	Yes
7. Were the outcomes measured in a valid and reliable way?	Yes	Yes	Unclear	Yes	Unclear	Yes	Yes	No	Yes
8. Was appropriate statistical analysis used?	Yes	Yes	Yes	Yes	Yes	Yes	Yes	Yes	Yes

de Carvalho, et al.	Diaz, et al.	Dufour, et al	Fernandes, et al.	Fokom Demgue, et al.	Gilkey, et al.	Goessl, et al	Hill, et al	Hoque, et al	Hurley, et al.
Yes	Yes	Yes	Yes	Yes	Yes	Yes	Yes	Yes	Yes
Unclear	Yes	Yes	Yes	Unclear	Yes	Yes	Yes	Yes	Yes
Unclear	Not applicable	Not applicable	Not applicable	Not applicable	Not applicable	Unclear	Unclear	Unclear	Unclear
Yes	Yes	Yes	Yes	Yes	Not applicable	Yes	Not applicable	Not applicable	Not applicable
Yes	No	Yes	Yes	Yes	Yes	Yes	Unclear	Yes	Yes
Yes	No	Yes	Yes	Yes	Yes	Yes	No	Yes	Yes
Unclear	Yes	Yes	Yes	Yes	Unclear	Unclear	Unclear	Unclear	Unclear
Unclear	Yes	Yes	Yes	Yes	Yes	Yes	Yes	Yes	Yes

Ishibashi, et al	Jaafar, et al	Jaspan, et al	Kahn, et al (2005)	Kasting, et al	Katsuta, et al.	Khamisy-Farah, et al	Killian, et al	Kubota, et al.	Lake et al.
No	Yes	Yes	Yes	Yes	Yes	Yes	Yes	Yes	Yes
Yes	Yes	Yes	Yes	Yes	Yes	Unclear	Yes	Yes	Yes
No	Unclear	Unclear	Yes	Yes	Yes	Yes	Yes	Yes	Yes
Not applicable	Not applicable	Yes	Not applicable	Not applicable	Not applicable	Not applicable	Yes	Not applicable	Not applicable
Yes	Yes	No	Yes	Yes	Yes	Yes	Yes	Yes	Yes
Yes	Yes	No	Yes	Yes	Yes	Yes	Yes	Yes	Yes
No	Unclear	Unclear	Yes	Yes	Yes	Yes	Yes	Yes	Yes
Yes	Yes	Yes	Yes	Yes	Yes	Yes	Yes	Yes	Yes

Lasset, et al	Lee, et al	Leung, et al.	Lutringer-Magnin, et al.	Mao, et al.	Maynard, et al.	McCave, et al	Merriel, et al.	Mohamed et al.	Napolitano, et al.
Yes	Yes	Yes	Yes	Yes	Yes	Yes	Yes	Yes	Yes
Yes	Yes	Yes	Yes	Yes	Yes	Yes	Yes	Yes	Yes
Unclear	Unclear	Not applicable	Yes	Not applicable	Yes	Yes	Yes	Yes	Yes
Not applicable	Not applicable	Yes	Not applicable	Yes	Yes	Yes	Not applicable	Not applicable	Not applicable
Yes	Yes	Yes	Yes	Yes	Yes	Yes	Yes	Yes	Yes
Yes	Yes	Yes	Yes	Yes	No	Yes	Yes	Yes	Yes
Unclear	Unclear	Unclear	Unclear	Unclear	Unclear	Yes	Yes	Yes	Yes
Yes	Yes	Unclear	Yes	Yes	Yes	Yes	Yes	Yes	Yes

Narayana, et al.	Nokolic, et al.	Nishioka, et al	Osaghae, et al	Petrusek, et al	Qaquish, et al.	Riedesel, et al.	Roland, et al.	Selvan, et al	Sherman, et al (2020)
Yes	Yes	No	Yes	No	Yes	Yes	Yes	No	No
Yes	Yes	Yes	Yes	Yes	Yes	Yes	Yes	Yes	Yes
Yes	Unclear	Yes	Not applicable	Yes	Not applicable	Yes	Yes	Yes	Yes
Yes	Yes	Yes	Yes	Yes	Yes	Not applicable	Not applicable	Yes	Yes
No	Not applicable	No	No	No	No	Yes	No	No	No
No	Yes	No	No	No	No	Yes	No	No	Yes
Yes	Yes	No	Unclear	No	Yes	Yes	Yes	Yes	Yes
Yes	Yes	Yes	Yes	Yes	Yes	Yes	Yes	Yes	Yes

Sherman, et al (2018)	Signorelli , et al,	Shuto, et al.	Song, et al.	Soon, et al.	Staras, et al.	Stamenkovic , et al.	Steben, et al.	Sypien, et al.	Thaker, et al.
Yes	Yes	Yes	Yes	Yes	Yes	Yes	Yes	Yes	Yes
Yes	Yes	Yes	Yes	Yes	Yes	Yes	Yes	Yes	Yes
Yes	Yes	Yes	Yes	Yes	Not applicable	Not applicable	Yes	Not applicable	Not applicable
Yes	Yes	Yes	Yes	Yes	Yes	Not applicable	Yes	Not applicable	Yes
Yes	No	No	Not applicable	Yes	Yes	Yes	No	No	Yes
Yes	Unclear	Unclear	Yes	Yes	Yes	Not applicable	No	No	Unclear
Yes	Yes	Yes	Yes	Yes	Yes	Yes	Yes	Unclear	Yes
Yes	Yes	Yes	Yes	Yes	Yes	Yes	Yes	Yes	Unclear

Tolentino, et al.	Tolunay, et al.	Wong, et al. (2016)	Wong, MC, et al. (2023)	Xu, et al.	Yetik, et al.	Yu, et al.	Yuta, et al.	Zhang, et al.
Yes	Unclear	Yes	Yes	Yes	Yes	Yes	Yes	Yes
Yes	Unclear	Yes	Yes	Yes	Yes	Yes	Yes	Yes
Not applicable	Not applicable	Not applicable	Not applicable	Not applicable	Not applicable	Yes	Yes	Not applicable
Yes	Not applicable	Not applicable	Not applicable	Not applicable	Yes	Yes	Yes	Yes
No	No	No	No	No	Yes	No	Yes	Yes
No	No	No	Yes	Yes	Yes	Yes	Yes	Yes
Yes	Unclear	Yes	Yes	Yes	Unclear	Yes	Yes	Unclear
Yes	Yes	Yes	Yes	Yes	Yes	Yes	Yes	Yes

Question	Agyei-Baffour, et al,	Kahn, et al (2007)	Krupp, et al.	Lasset, et al	Lutringer-Magnin, et al	Moya, et al	Tron, et al.
1. Is there congruity between the stated philosophical perspective and	Yes	Yes	Yes	Yes	Yes	Yes	Yes
2. Is there congruity between the research methodology and the research	Yes	Yes	Yes	Yes	Yes	Yes	Yes
3. Is there congruity between the research methodology and the methods	Yes	Yes	Yes	Yes	Yes	Yes	Yes
4. Is there congruity between the research methodology and the	Yes	Yes	Yes	Yes	Yes	Yes	Yes
5. Is there congruity between the research	No	Yes	Yes	Yes	Yes	Yes	Yes
6. Is there a statement locating the researcher	No	Yes	No	No	No	No	Yes
7. Is the influence of the researcher on the research, and	No	Yes	No	No	No	No	Yes
8. Are participants, and their voices, adequately	Yes	Yes	Yes	Yes	No	Yes	Yes
9. Is the research ethical according to current criteria or, for recent studies, and is there evidence of ethical approval by	Yes	Yes	Yes	Yes	Yes	Yes	Yes
10. Do the conclusions drawn in the research report flow from the analysis, or	Yes	Yes	Yes	Yes	Yes	Yes	Yes
