# Supplementary material for: Healthcare worker practices for HPV vaccine recommendation: A systematic review and meta-analysis
Source: Hum Vaccin Immunother. 2024 Oct 14;20(1):2402122. doi: 10.1080/21645515.2024.2402122 (PMC11486212; doi:10.1080/21645515.2024.2402122)
Supplement: Appendix 6_Potential facilitators of HCW recommendation.docx [file KHVI_A_2402122_SM6234.docx]

**Potential Facilitators of HCW Recommendation for the HPV Vaccine**

| **Characteristic** | **Studies Reporting Association (References)** | **Direction of Association** |
| --- | --- | --- |
| Provider Gender | **19/21** (24, 25, 27, 29-31, 44, 49, 65, 68-71, 76, 78, 82, 84, 85, 91) | Female providers more likely to recommend |
| Knowledge Scores | **17/17** (24, 25, 27, 42, 44, 57, 70, 74, 75, 78, 80, 83, 85, 91, 96) | Higher knowledge scores associated with willingness to recommend |
| Provider Age | **10/13** (25, 29, 38, 40, 48, 50, 60, 64, 65, 69, 71, 74, 78, 82, 84, 85, 88, 96) | Younger providers more likely to recommend |
| Provider Specialty | **8/9** (38, 50, 60, 69, 71, 74, 78, 85) | OB/GYNs more likely to recommend than other specialties |
| Practice Location | **4/4** (34, 47, 73, 82) | Urban providers more likely to recommend than rural providers |
