# Supplementary material for: Healthcare worker practices for HPV vaccine recommendation: A systematic review and meta-analysis
Source: Hum Vaccin Immunother. 2024 Oct 14;20(1):2402122. doi: 10.1080/21645515.2024.2402122 (PMC11486212; doi:10.1080/21645515.2024.2402122)
Supplement: Appendix 3_Summary table of included articles.docx [file KHVI_A_2402122_SM6233.docx]

Table 1. Summary table of included articles synthesizing author, year of publication, study location, study aim, population, methods, recommendation sentiments and barriers.

| **Authors and Year** | **Location** | **Study Aim** | **Population** | **Methods** | **Recommendation Sentiments** | **Barriers** |
| --- | --- | --- | --- | --- | --- | --- |
| **Abi Jaoude, et. al, 2019** | Lebanon | (1) Identify barriers, attitudes and clinical approaches of physicians towards HPV vaccine, (2) analyse associations b/t physician demo and characterizes and barriers to HPV vax | Physicians of OBGYN, Paediatrics, Family, and ID from Lebanese order of Physicians | Cross sectional study with survey gathering demographics, knowledge, and vaccination barriers. Physicians called to participate and offered face to face, phone or email survey administration. | ~25% always recommend for females, but only 6% for male patients | Barriers include cost of HPV vaccine, failure of insurance companies to cover cost, vaccine safety and efficacy. 45.9% considered the HPV vaccine not being mandatory a barrier. Both parents (73.8%) and physicians (49.8%) present barriers related to discussing sexuality and STDs. |
| **Agyei-Baffour, et. al, 2020** | Ghana | Examine factors that influence HCPs general practices towards HPV and perceptions of factors that could engage parent or adolescent HPV vaccination | Physicians, nurses, hospital admin | Qualitative analysis strategy using focus group discussions. Used a purposive sampling strategy to get a diversity of perspectives. Conducted 3, 60-minute focus group discussions. Did a directed content analysis. | Rarely offered HPV vaccination recommendation | Competing priorities, lack of awareness and evidence to support efficacy and safety, and/or unavailability of resources (immunization kits). |
| **Almughais, et. al, 2018** | Saudi Arabia | To evaluate PHC physicians' awareness of HPV infection and vaccination and assess their perception towards recommending HPV vaccine in Saudi Arabia. | Primary healthcare physicians | Self-administered survey assessing knowledge of HPV infection, vaccines, perceived importance of vaccines, and if they would recommend the vaccine to patients. | 16.5% recommended the vaccine to patients. Females 3.1 times more likely to recommend. | N/A |
| **Albayat, et al. 2024** | Qatar | The study object was to assess the knowledge, attitudes, and practices among physicians working in Qatar. | Physicians | Cross-sectional study design with a pretested online survey tool with four sections; demographics, knowledge, attitudes, and practice. A mean knowledge score was calculated and then using bivariate and multivariate analysis they looked at association between knowledge and attitude/practice. Logistic regression for predictors for recommending the HPV vaccine. | More than one third were NOT interested in recommending | Perceived barriers to community acceptance were lack if awareness regarding relationship between HPV and cervical cancer, doubts regarding efficacy/safety, encouraging risky sexual behaviour, low risk, and cost. Muslim providers less likely to recommend. |
| **Ayash, et al. 2023** | USA | This study explores providers' perceptions, barriers, and experiences related to HPV vaccine recommendation and uptake among Arab American patients. | Physicians, nurse practitioners and PAS. | Conducted a cross-sectional survey among providers survey adolescents and young adults from the greater New York City area, who had a greater than 5% Arab American practice population. An existing survey from Luque et al 2014 was adapted to determine HPV vaccine recommendation practices. Survey developed from the Competing Demands Model. Descriptive statistics for survey responses and thematic analysis for written responses. | 78% were comfortable suggesting the HPV vaccine when a patient came in for an unrelated problem. | Cultural concerns around increasing risk behaviour, cost/lack of reimbursement, patient embarrassment or concerns. |
| **Ayres, et. al 2021** | United States of America (USA) | Study compared HPV vaccination occurrences on the same day as provider recommendations at clinics in the US by healthcare provider groups. And describe providers perceived challenges, HPV vax knowledge, and HPV recommendation practices, | Paediatric healthcare providers and staff | Participants were divided into two groups: clinicians (medical doctors, residents, PAs, nurse practitioners) and other healthcare team members (OTM, nurses, MA, clinic staff, admin, stakeholders). 40-question survey administered with demographics, perceived challenges scale, knowledge scale, a recommendation practices scale, same-day HPV vaccination question. | Clinicians more likely to recommend to boys and girls 11-12 than OTMs. Confirmed association between strong recommendation practices and same-day vaccination (driven by OTMs) | Clinicians had more challenges than Other medical team member. |
| **Btoush, et. al, 2022** | USA | Examine factors associated with healthcare providers (HCPs) recommendation of HPV for younger and older adolescents. | Physicians, nurse practitioners, paediatrics, family medicine, and women's health) | Cross-sectional study with web-based survey measuring HCPs recommendation of the HPV vaccine with practice characteristics and HCPs characteristics. 60-item survey that assessed knowledge, beliefs (effectiveness and concerns), and perceived barriers and facilitators. | Recommendation rates for "always" (>75% of the time) recommending HPV were 56% for younger adolescents (11-13 yrs) and 73% for older (14-17 yrs). Key drivers were paediatric speciality and HPV vaccination knowledge. | System level barriers (ordering, stocking, admin) and barriers to communicating with parents about the vaccine. HCPs noted safety, long term effects, drug companies pushing for money, experimenting on children as concerns, as well as the vaccine discouraging routine pap smears, increasing sexual promiscuity, and decreasing condom use. |
| **Chawla, et. al, 2016** | India | Explore knowledge, attitude, and practice of healthcare providers in India. | Healthcare professionals | Cross-sectional study from questionnaire data collection of public hospitals, private hospitals and PHCs. Self-administered validated questionnaire to assess for KAP for HPV and HPV vaccination. | Only 47% of respondents recommended young women to get vaccinated against HPV, but 76% of gynaecologists recommending with only 4% of paramedical staff doing so. | N/A |
| **Chen, 2022** | China | Aims to assess awareness, knowledge of HPV and HPV vaccine as well as recommendation behaviour towards vaccination among HCPs in Southern China | HCWs | Large-scale cross-sectional survey across 3 Chinese providences. Randomised cluster sampling with target of 2000 responses. Questionnaire consists of 55 questions with 4 domains (socio-demographic info, awareness of HPV and vaccine, knowledge, and willingness and behaviour of recommending vaccine). | 94.8% willing to recommend to others. 68.1% reported having recommended HPV vaccine to others. | Main reason for unwillingness to recommend was safety of the vaccine. |
| **Daley, et. al, 2010** | USA | Assess among paediatricians and family physicians, HPV vaccination practices, perceived barriers, and factors associated with recommending HPV vaccine. | Paediatrics and family Physicians | Survey given to national network of PCPs. Attitudes and perceived barriers measured on Likert scale, while HPV practice and experiences with vaccine refusal were only asked to those administering. | Strongly recommending the vaccine varied based on patient age. For 11-12 yr olds, 56% of peds strongly recommend and 38% recommend. Family physicians recommend it was 50% and 43%. | Most frequently reported were financial (insurance coverage, reimbursement, and vaccine purchasing costs). 23% of paediatricians and 33% felt parental opposition for moral or religious reasons was definitely or somewhat of a barrier. Also reported parental concerns about safety, but the physicians themselves rarely reported concerns about safety. |
| **de Carvalho, et. al, 2009** | Brazil | Primary objective to evaluate physicians and medical student acceptability and knowledge of HPV vaccine | Medical students, OBGYN, paediatricians, and other doctors. | 13 question survey regarding the vaccine and gathered personal information. Included intention to prescribe and to what age, gender, and common knowledge regarding HPV vaccination. | 79.7% declared that they would prescribe vs 20.3% who said they would not or did not know. Not statistically significant difference between groups. | 12.2% still sceptical about the vaccine providing lasting immunity. Some also concerned about adverse side effects (more students than doctors) |
| **Diaz, et al, 2023** | USA | To determine the knowledge and comfort in discussing the HPV vaccine among physicians in South Carolina. | Paediatricians and Family medicine providers | A cross-sectional anonymous survey that was developed and validated with an expert panel. Survey questions on current clinical practices related to HPV, participation in the Vaccines for Children program, availability of onsite HPV vaccination, geographic data point, provider knowledge, self-rank their knowledge, comfort level discussing HPV, and ability to provide a strong endorsement of the vaccine. | 98.2% of respondents strongly endorse the HPV vaccine and 96.4% endorse childhood vaccines. (interesting that it is higher) | N/A |
| **Dufour, et. al, 2023** | France | Assess the knowledge, attitudes, and practices regarding HPV vaccination among pharmacists, particularly in boys. | Pharmacists | Cross-sectional, quantitative, and descriptive survey. A 46 question- questionnaire both closed and open questions. Ordinal logistic regression with cumulative link models performed for determinant levels of knowledge with covariates of fender, profession, # of years of practice and pharmacology. Binary logistic regression with linear model to determine right attitude towards HPV vaccination. | 69.8% said they have advised girls, 34.0% for boys, and 7.4% for MSM. | Reasons for not advising were lack of opportunity or did not think to do it. Other reasons were it was complicated to discuss (esp with parents and MSM), lack of information, recommendations for boys is too recent, it’s the physicians role, and current focus is on COVID-19 vaccination. |
| **Fernandes, et al., 2023** | USA | Aim to describe HPV vaccine knowledge, attitudes, and recommendations/discussion practices. | Medical providers, dentists, and pharmacists. | This was a prospective descriptive study with a self-administered survey that was pilot tested. Gathered data on HPV vaccine knowledge, HPV vaccine attitudes with ordinal scale, recommendation practices (frequency and age). Used categorical variables to summarize frequences and proportions. Pearsons correlation for statistical correlation, and Chi-squared test. | Just over half of medical providers, less than a third of dentists and fewer than a fifth of pharmacists report recommending the HPV vaccine. | N/A |
| **Fokom-Demgue, et al, 2024** | 23 countries in Africa | Assess the training, knowledge, and recommendation of the HPV vaccine among African providers in cervical cancer prevention. | Healthcare providers involved in cervical cancer prevention. | Cross-sectional study conducted among health care providers involved n cervical cancer prevention activities in Africa. They reported (prior to and post a training) the availability of HPV in their practice, whether they recommend HPV or not, and reasons for not recommending. | Majority (83.2%) reported that they were recommending HPV vaccine in their practices. Recommendation was higher in country settings with an NIP (88.3% vs 73.9%) | Vaccine unavailability (57.1%), lack of effective communication tools and informational material (28.6%), and need for adequate training (28.6%) were the most commonly reported reasons for not recommending the HPV vaccine. These barriers were higher in countries without NIPs. |
| **Gilkey, et al, 2015** | USA | Sought to describe HPV vaccine communication practices among primary care physicians. | Primary care physicians | Through a national online survey they assess the quality of HPV vaccine recommendations based on strength, timeliness, and urgency. Used a developed framework from national practice guidelines and a lit review, that is focused on timeliness, consistency, urgency, and strength of endorsement. Combined this into an overall recommendation quality (low quality and high quality). | Recommend the vaccine on time for males is 74% (573) and females 61% (472). Strength of endorsement as very/extremely important is 568. | N/A |
| **Goessl, et. al, 2022** | USA | Objective was to use multivariable modelling to identify the HPV vaccine attitudes and practices that were most strongly associated with rural vs urban providers. | Adolescent care providers (rural vs urban) | Cross-sectional survey to all licensed adolescent care providers in counties in the two states. Survey contained 28 items covering the outcome measures including a section of recommendation strength and frequency for HPV vaccines and other adolescent vaccines. Multivariate logistic regression to identify attitudinal and practice exposure in rural vs urban. | Fewer rural providers (12%) routinely recommended HPV vaccine during acute care visits than urban providers (22%) (aOR=0.37), but had more standing orders to administer recommended adolescent vaccines (77% vs 51%) (aOR=2.81) | N/A |
| **Hill, et. al, 2014** | USA | Assess willingness of emergency medicine (EM) physicians to recommend the vaccine, target high-risk women, and disclose perceived barriers to vaccination. | ED Physicians | 10-item survey with some pre-validated survey questions and some new items. All ED faculty or residents at a Texas hospital. Independent variables were comfort with HPV vaccination and targeting high risk women, HPV related beliefs about vaccines, need based beliefs to test HPV prior to vaccination or discussion sexual behaviour prior. | 67.4% would recommend the vaccine, 23.9% were neutral, 8.7% would not recommend to age eligible patients. | 41% notes lack of adequate reimbursement as barrier to vaccination. Other barriers assessed were time related (70.3% strongly agree or agree) and reservations about ED HPV vaccination due to the inability to assess completion of vaccination series (82.6%). |
| **Hoque, et. al, 2014** | South Africa | Determine factors contributing to recommending vaccination to patients among nurses at a tertiary hospital in SA. | Nurses | Cross sectional study focusing on nurses from a hospital. Self-administered questionnaire used was one developed by Walsh and McPhee, which is a model that considers HCWs predisposing factors, knowledge as enabling, and professional and social support as reinforcing. Four sections of questionnaire: general info, HPV infection and prevention, HCWs practice related characteristics, and demographics. | 90.9% intended to recommend the vaccine | N/A |
| **Hurley, et. al, 2021** | USA | Objectives were to assess PCPs recent vaccination practice in adults, knowledge of HPV and recommendation, and attitudes towards recs | Primary Care physicians | Survey administered to national network of PCPs in the US. Ask specifically about patients aged 27-45 years of age. Frequency of patients requesting HPV vaccine, and how often the physician gave it. True and false questions on knowledge. Likert scale for attitude. How will the official recommendations affect their behaviour. | 42% had recommended to adults 27-45 in the past. In the past 3 months, 73% had not administered to this age range. | N/A |
| **Ishibashi, et al, 2008** | USA | Examined whether paediatricians would recommend the vaccine, obstacles encounters and characteristics associated with not recommending. | Paediatrics physicians | In 2006 they surveyed 850 paediatricians on opinions on the new HPV and rotavirus vaccines. Survey included physicians attitudes and intended practices for incorporating HPV and rotavirus into immunization. Also asked what factors influenced decision not to recommend (safety, cost, increased sexual activity, or other). | 88% would give to all their patients. | 21 concerned about cost, 10 about safety and 5 about that it might lead to increased sexual activity. Other reasons were age too young, concerned about reimbursement, undecided and not needed for some. |
| **Jaafar, et. al, 2022** | Lebanon | Assess the change in knowledge, awareness, and attitude (KAA) of Lebanese HCPs or OBGYN who attend educational meetings, symposia and workshops from 2009 and 2018, and identify factors affecting attitudes. | HCPs (Mostly OBGYNs) | Standardise, anonymous questionnaire that were measured in 2009 and 2018. Survey included questions related to knowledge, pathogenesis of HPV diseases, characteristics of the vaccine, and attitudes towards HPV-V and relevant issues in discussing with patients. | About 20% usually recommend HPV. But the majority (69 and 74%) discussed HPV and HPV-V with patients. More in 2018 recommended gender neutral vaccination. | 20% of those who usually recommend HPV report cost as a main barrier and 76% of those who do not regularly recommend reported cost as a main barrier. Other barriers reported by recommenders were refusal to discuss sexual issues, fear of adverse events, false perception of low risk. |
| **Jaspan, et. al, 2008** | USA | Determine the percentage of patients vaccinated per provider and assess attitudes and reasons for accepting HPV vaccine | OBGYNs | In a Philadelphia medical centre group of OBGYNs. Departmental policy created to give patients info and offer the vaccine along with continuing medical education on the vaccine for physicians. Vaccine administration data was gathered from computerised system. Questionnaire distributed for assessing physician attitudes. | Vaccination rates varied from 6 to 55.8% by provider. 5 had vaccination rates under 20% and 4 had vaccination over 20%. | 33% identified patient concerns to safety and cost as barriers. 33% said patients remained uncertain after discussion in office, 33% identified cost as a personal concern. |
| **Kahn,et. al, 2007** | USA | Describe paediatricians’ attitudes about HPV vaccines and explore factors influencing intention to recommend | Paediatricians | Semi structured individual interviews and framework analysis was used. Participants completed a survey and then an interview was conducted with open-ended, standardised questions. End point of the analysis was creation of a conception framework with categories and subcategories generated from the data. | 27 (87%) were somewhat or extremely likely to recommend the bivalent vaccine and 29 (94%) for the quadrivalent vaccine. High intention to recommend overall but varied by patient age and gender, and HPV vaccine type. | Barriers were lack of parental knowledge about HPV and parental denial that child is sexually activity and at risk of HPV. Lack of provider knowledge as well. Concerns that administering the vaccine will encourage risky behaviour. Paediatricians who were not likely to recommend the vaccine cited a lack of information on safety and efficacy as main decision making factors. |
| **Kahn, et. al, 2005** | USA | Examine paediatricians’ characteristics and attitudes associated with intention to recommend two hypothetical HPV Vaccines | Paediatricians | Mailed out the survey twice to 1000 paediatricians. Survey is grounded in the Theory of Planned behaviour, which says that the most important determinant is intention to preform that behaviour. Two primary outcomes were intention administer cervical cancer vaccine and a cervical cancer/genital warts vaccine. | Mean intention to vaccine varied from 4.74/5.0 for cancer/genital warts for girls age 17 to 2.80/5 for cancer vaccine for boys of 11. Preference for combined cervical cancer/genital warts vaccine and for vaccinating girls, and generally more likely to recommend for older rather than young patients. | Main barriers were about parental barriers such as parental vaccine safety concerns and reluctance to vaccinate a child against an STI. |
| **Kasting, et. al, 2021** | USA | Evaluate OB/GYN knowledge, attitudes and beliefs regarding HPV vaccination for younger and mid-adult women | OBGYNs | Survey of OBGYNs practicing in the US and used previously developed tools where possible. Consisted of 73 questions. Asked about HPV recommendation frequency, strength, and consistency. Knowledge assessed with 7 true/false questions and summed to create a knowledge score. Participants were given 9-items scale to measure importance of certain items in recommending, benefits measured in 4 items, and barriers with 8 for both age groups. | 84.2% recommend the HPV to age eligible patients usually or always but perceive only about 50.7% of their colleagues recommend. More frequently recommend to patients 19-26 compared with those 27-45. | More barriers for older age group. Most frequently reported barriers are cost, regardless of age. More than 1 in 10 had concerns about vaccine safety for 19-26 and more for older age. |
| **Katsuta et al, 2019** | Japan | Examined attitudes and intentions of Japanese physicians relating to adolescent vaccines, specifically HPV | Paediatricians, internists, and OB/GYNs | A cross-sectional survey was mailed to physicians. Included questions about frequency of providing education, two questions on sexual health, 3 questions relating to physicians perceptions of vaccine recommendations, vaccine safety and vaccine effectiveness, and if they would recommending with the ministry reinstated the recommendation. Categorized frequency of education and have a recommend actively or if requested as an HPVV acceptance group. Then used Ryans method for multiple comparisons. | 44% of physicians would recommend actively if the MHLW reinstated recommendation, and 45% would if requested by patients/parents (total of 90%). | Safety, lack of ministry proactive recommendation. |
| **Khamisy-Farah, et. al, 2019** | Israel | To investigate knowledge of HPV and HPV related issues, and attitudes and practices towards recommending HPV vaccine. Knowledge, Attitude, and Practice (KAP), questionnaire developed and validated in a sample | Paediatricians, gynaecologists, and internal med docs | KAP questionnaire was developed from a lit review, a focus group of HCWs, revision from an expert panel, a pilot test with 20-30 subjects. Consisted of 3 parts; sociodemographic information, knowledge of HPV and burden, and attitudes and practices | 98.6% recommended to girls and 79.9% recommended to boys. | N/A |
| **Killian, et. al, 2016** | France | Gain knowledge about vaccine hesitancy among GPs by comparing their attitudes towards vaccination for themselves, their children, and their patients. | General Practitioners | Completed a web-based survey sent to private practices. Checklist for Reporting Results of Internet E-Surveys was followed where possible. For each vaccine, the GPs were asked if they recommend to their patients and if they had followed official guidelines for their children. Recommendation guidelines from French authorities used as reference. | HPV recommendation <75% for their daughters and <85% for patients. ~1/4 of GPs who are given children HPV vaccine would use an alternative schedule. | Barriers include religious beliefs, parents concerns about effect on children's sexual behaviour, and low perceived risk of HPV. |
| **Krupp, et al. 2010** | India | Investigates physician intention-to-recommend the HPV vaccine to parents of adolescents girls in India. | Physicians (broad range of specialties) | Conducted 20 in-depth interviews with physicians from the Mysore district. Referred from public health institute and then snowball sampling. Interview guide was created from a literature review. For data analysis conducted an iterative analysis process to find emergent themes. | 18 (90%) stated that physicians should recommend the HPV vaccine. | Limited information on the vaccine efficacy and safety. High cost fo the vaccine for patients. Parental concerns about sexual activity. Lack of time to counsel patients. Unable to recommend vaccines not yet part of the universal vaccine program. |
| **Kubota et al, 2022** | India | Measured change in attitudes of paediatrician regarding the HPV vaccine between 2020 and 2021. | Paediatrician | Sent the same electronic survey out in 200 and 2021. Questionnaires asked about status, intentions about HPV vaccination, how they acted with a child of target age, general impressions of the public, and concerns about HPV vaccine. | In 2020, 78.5% provided between 1-10 and 14.0% provided between 11-30. In 2021, this was 59.6% and 29.4% respectively. | Safety concerns relating to symptoms mentioned by media, and injection site concerns. |
| **Lake, et al. 2023** | USA | Describes HPV vaccine recommendation practices among clinicians in federally qualified health centres. | Clinicians, NPs, CNMs, PAs, and others. | Survey based on was based on the impact of COVID-19 on cervical cancer screening. The survey was reviewed by experts and refined based on their feedback. Asked how they recommend HPV vaccines for ages 9-10, 11-12, 13-18,19-26, and 27-45. Response options included strong, offer but not strong, recommend against, unless patient bring it up, I do not see this age range in my practice. Used descriptive statistics for characteristics and outcome variables. binary logistics regression for factors associated with HPV vaccination to ages 9-10. | For ages 9-10, 65% strongly recommend (80), 23% offer but not strongly, 11% do not discuss unless brought up by patient. | N/A |
| **Lasset, et. al, 2014** | France | Investigate the evolution of practices and opinions regarding the HPV vaccine in French GPs. | General Practitioners | Previous cross-sectional study from 2007 (Lutringer-Magnin, et. al, 2007), which assesses HCW and HPV vaccine. Repeated here in 2010. Used both quantitative and qualitative methods. Compared quant results across the two years. Qualitative approach was exploratory design. Self-questionnaire used to gather general info, opinion about HPV vaccine, practice, difficulties encountered, and opinions regarding opportunities and inconveniences created by HPV. | 82.7% (2010) reported having delivered the HPV vaccine in the prior month and 49.1% have frequently vaccinated 14 yr old girls, which is higher than previous study (75.6 and 19.0). No difference at 15-23 yrs age group (67%) | Both years about 56.7% reported at least one complication related to vaccination, but barrier questions asked by patients decreased. Reason for medical consultation increased as a difficulty. Necessity to address STIs was only identified as a problem for 6.2% in 2007 and 13.4% in 2010. In qual, the main concerned that remained was side effects, which were difficult to address with patients. General fear expressed by patients. Some felt an older age might be appropriate. |
| **Lee, et. al, 2019** | Hong Kong | Investigate practice of recommending HPV vaccine to attendees among providers at public health clinics in Hong Kong | Medical doctors and nurses | Used the Theory of Planned Behaviour model as a study framework. A cross sectional survey was conducted to investigate recommendation practices and associated factors. Socio-demographics, recommendation of HPV vaccination to STD clinic attendees, knowledge related to HPV vaccine, and perceptions recommending HPV to STD clinic attendees (4 scale based on TPB). | 16.3% recommended to males, 36.7% recommended to females, 41.8% to either. | N/A (article discusses that embarrassment to talk about sex topic and perception of HPV vaccine as a symbol of promiscuity were barriers to HPV vaccine uptake--thus use of STD clinics) |
| **Leung, et al. , 2023** | USA | Object was to create a 7 min interactive online educational tool to improve knowledge and willingness to increase HPV recommendation among nurses. | Nurses | A prospective pre- and post- test study to evaluate the effectiveness of an educational tool with 10 flashcards in a question-answer format. Oncology nurses at the cancer centre invited to participate by email. Pre- and post- survey gathered information on HPV-associated cancers, vaccine eligible age groups, dosing schedule, adverse events, and willingness to recommend. The educational intervention was 10 interactive flashcards with the final one directing them to a CDC video on How I Recommend to provide practical advice. | At baseline 93% would recommend the vaccine to patients. | N/A |
| **Lutringer-Magnin, et. al, 2011** | France | Examine the perceptions, attitudes and practices of GPs regarding HPV vaccination. Increase understanding of the factors influencing GPs. | General Practitioners | Mixed methods cross sectional study design with self-administered questionnaire and interviews. Survey gathered information on general info, data on their practice regarding certain vaccines, attitudes towards prevention of smoking, obesity, cardiovascular risk factors, and cervical cancer screening, along with practice, attitude, opinion in relation to HPV vaccine. 35 chosen with favourable and unfavourable HPV vaccine opinions for interviews. Explored knowledge, French recommendations, beliefs, reasons for opinion, and difficulties encountered in providing it. | Month before the survey, 75.6% had given the HPV vaccine, and 47.6% had given it at the recommended age of 14 and 67.7% for 15-23 (36.9% only vaccinated the old age group). | 43.4% of the uncertain or opposed cited the too recent introduction of the vaccine. Only 8 said reasons related to the adequacy of cervical screening. Major difficulties were patient concerns about side effects and the target age of 14 (interviews suggest related to not wanting to discuss STDs with adolescents). |
| **Mao, et al. 2023** | China | Aim of this study was to assess the frequency of HCP recommendations for HPV vaccination and associated factors. | Physicians and nurses who worked in obstetrics and gynaecology,  preventive health care, paediatrics and general medicine departments | A cross sectional study through online questionnaires from hospitals in three large cities in China. Questions involving demographic information, frequency of HPV vaccine recommendation, knowledge of HPV and attitudes towards HPV related topics. Frequencies and proportion reported for HCPS HPV vaccine recommendations. IQRs for knowledge and attitudes. Then Chi-square tests used for demographic characteristics and HPV recommendation frequency. | 30.2% reported that they frequently recommend the HPV vaccine. | Mid-level concern regarding non-obligation to recommend, concern of being seen as hard seller, low risk of disease, vaccine sceptics, and difficulty communicating regarding sexual topics. HCPs who perceived no obligation to recommend or experienced difficulty discussing sexual health topics had decreased frequency of recommendation. Many also thought that parents had concerns regarding vaccine safety and efficacy. |
| **Maynard, et al., 2024** | USA | This paper aims to describe the implementation and initial outcomes of the training provided. | Nurses, medical assistant, physicians, and advanced practice providers. | Implemented a 20 minute training called Communicating about HPV vaccination to Adults and Teens (HPV CHAT) program focusing on HPV vaccine. They integrated training with clinical care teams and assessed immediate training outcomes compared with pre-training. Descriptive statistics used to summarize pre and post survey responses. | In the pre-test 128/187 (68.4%) plan to routinely recommend the HPV vaccine. And 137/187 (73.3%) plan to or already recommend the HPV vaccine to patients. | N/A |
| **McCave, et. al, 2010** | USA | Examine providers perceived barriers, supports, and vaccination actions in delivering the HPV vaccine to females 9-17 in four states. | Paediatricians and family Physicians, gynaecologists, nurse practitioners, and physician assistants | Cross sectional survey with primary care providers. The states included were New Mexico, North Carolina, Texas, and Louisiana. Survey was created and piloted for validity. Survey covered professional and demographic info, questions regarding vaccination for 9-12- and 13–17-year-olds, providers HPV vaccination actions, perceived barriers, and supports. TPB as foundation. | HPV vaccination rates are significantly higher for girls 13-17 than 9-12, across all four states. | Financial burden of HPV vaccine and patients (and parents) who have negative perceptions of the vaccine |
| **Merriel, et. al, 2018** | United Kingdom | Aimed to explore and compare the knowledge and attitudes of UK GPs and sexual health professionals regarding HPV vaccination of young MSM (16-24) | General Practitioners and sexual healthcare professionals | Cross sectional online questionnaire as part of a mixed methods study. Questionnaire informed by steering groups with LGBTQ stakeholders and MSM sexual health researchers and piloted prior to wider distribution. Adapted from HCW HPV attitude scale and HCP PrEP scale. | 22 had vaccinated MSM. Sexual health providers are more likely to have vaccinated MSM than GPs. | Factors preventing vaccinating MSM were lack of time (65.79%) (biggest for GPs) and staff availability (55.10%) (biggest for sexual health providers). Also concerns about vaccine availability. |
| **Mohamed et al, 2023** | Egypt | Assess the knowledge, attitudes, and practices of cervical cancer prevention (pap smears and HPV vaccine)among OB/GYNs | OB/GYNs | Using a cross-sectional study design, all ob-gyns at a conference completed the survey. Questionnaire included questions on demographics, work experience knowledge, attitudes and practice of pap smears and HPV vaccine using Likert and multiple choice. Followed the KAP framework. | 60% would recommend it to their patients. | N/A |
| **Moya, et al., 2023** | USA | As part of a wider intervention project, this study aims to use focus group discussions to inform the culturally-tailored, bilingual, intervention to increase HPV vaccine uptake, screening and critical health literacy. The aim of this focus group is to understand perceptions about the roles of CHWs and HCPs in increasing HPV-vaccine uptake. | Physicians, nurses, nurse practitioners, and Community health workers. | A qualitative study with three focus groups with CHWs and HCPs and they completed a brief questionnaire. The initial survey gathered demographic information, and included questions on their HPV knowledge, attitudes and practice. Independent codes conducted content analysis to identify themes from the FGDs. | All stated that they would recommend the HPV vaccine. For self-perceived roles in HPV cancer prevention CHWs had no mention of recommending HPV vaccine and HCPs felt their role was to make "strong recommendations" to get the HPV vaccine. | Some of the perceived obstacles to effort to prevent HPV cancers were patients financial situations, knowledge of HPV, influence of patriarchy, and attitudes. Non-patient related obstacles included high costs of HPV vaccines, vaccine accessibility, and inconsistency in practices. |
| **Napolitano, et. al, 2018** | Italy | Assess primary care paediatricians (PCPs) attitudes and practices regarding HPV. | Primary Care, and paediatricians | Cross sectional study design with online questionnaire using multi-stage sampling from local health units. Questionnaire was pretested and piloted. 46 items to cover the four main areas. | Overall, 98.7% recommended to girls rarely to always. 77.4% always recommend the HPV vaccine to girls 11-12. Overall, 58.9% recommend the HPV vaccine to boys rarely to always. Only 18.4% were always recommending it to 11–12-year-old boys. | The HPV vaccine not being actively recommended in their region (88.7% of those not always recommending), lack of time, and concerns about efficacy or side effects were barriers among Boys. For girls reasons were lack of time, concern that vaccination could increase high risk behaviour, and concerns about safety and efficacy. |
| **Narayana, et. al, 2020** | India | The aim of the study is to evaluate the Knowledge, Attitude, and Practice (KAP) of physicians in the recommendation of HPV vaccination. | Physicians (OBGYN, paediatricians Oncology, Family Medicine, Infectious Diseases) | The method used for this study was a ​cross-sectional design. With a survey that included adequacy of Knowledge, Attitude, and Practice towards HPV vaccine recommendation. | Only 33.4% of physicians were recommending the HPV vaccine to their clients. | The study identified barriers to HPV vaccination like vaccine side effects, cost, doubts about vaccine efficacy, concerns about sexually transmitted diseases (STDs), safety concerns, and non-availability of the vaccine in the Universal Immunization Program (UIP). |
| **Nikolic,et. al, 2015** | Serbia | The aim of the study is to investigate the factors influencing the recommendation of the human papillomavirus (HPV) vaccine by Serbian paediatricians. This includes examining their knowledge, attitudes, and intentions regarding the HPV vaccine. | Paediatricians | The method used for this study was a ​cross-sectional design assessing nurses' knowledge, attitudes, and practices related to human papillomavirus (HPV) vaccination via a survey with five parts. | Nearly two-thirds of the paediatricians (60.2%) in Serbia are willing to recommend the HPV vaccine. | Barriers are Underestimation of HPV risk, Challenge of completing a 3-dose regimen, Long-term safety concerns, Concerns about cost, Parental barriers to acceptance, Erroneous claims about the connection between vaccines and mental retardation. |
| **Nishioka, et. al, 2022** | Japan | The aim of the study was to assess healthcare workers' recommendations regarding HPV vaccination and examine how providing information about the vaccine influenced their recommendations. | Nurses and Physicians | A cross sectional study design with a questionnaire that asked about HCWs understanding, recommendations, and opinions regarding HPV vaccination. | Only 19% of healthcare workers reported always recommending the vaccination. | The barriers to HPV vaccination identified in the study include: Low recommendations from healthcare workers. Concerns about adverse events reported by the media. Lack of active recommendation from the Ministry of Health, Labour, and Welfare. Insufficient information and awareness about the risks and benefits of HPV vaccination |
| **Osaghae, et al., 2023** | USA | To assess the determinants and barriers to consistent offering of HPV vaccine among healthcare facilities | Healthcare providers | This was a cross-sectional survey of healthcare providers (HCPs) in Texas. The survey assessed for determinants and barriers to consistent offering of HPV vaccine among healthcare facilities. Compared using odd ratios. |  | HPV vaccination not within the scope of the practice (48.1%), referrals to other clinics (27.7%), and limited personnel (11.4%) |
| **Petrusek, et. al, 2020** | USA | The aim of the study is to examine HPV vaccine administration practices since FDA approval up to age 45 and assess knowledge regarding HPV and its association with oropharyngeal cancer. | General Practitioner | The method used in the ​study is a ​cross-sectional descriptive with an 11-question survey to assess HCW HPV vaccination practices, knowledge of FDA approval, and barriers to vaccination. | The study found that more than 95% of the surveyed primary care physicians recommended HPV vaccination for males ages 9-21 and females ages 9-26. However, the recommendation rates decreased significantly for males older than 21 and females older than 26, with only 52% and 35% of physicians recommending vaccination, respectively. | According to the survey, the primary barriers to HPV vaccination reported by physicians were negative perception of the vaccine by patients and parents. Other barriers mentioned included conflicting information online, issues with insurance coverage, and specific concerns raised by patients or parents. |
| **Qaqish, et al., 2023** | Jordan | To investigate the knowledge and awareness of Jordanian physicians on such routes. | Physicians | A questionnaire was conducted among a national Jordanian sample of physicians from Jordanian health sectors. The survey included questions assessing participants’ knowledge on HPV, non-sexual routes of infection and HPV vaccines. Physicians’ attitudes towards HPV screening and vaccination were covered. | 331 (82%) stated that they will recommend the HPV vaccine to their patients if the vaccine was available in Jordan for free, while 72 (18%) won’t. | Barriers cited include cultural reasons, and vaccine not safe or not protective |
| **Riedesel et al, 2005** | USA | Examine the attitudes about HPV immunization among family physicians with intention to immunize. | Family Physicians | Sent out survey to physicians from American Academy of Family Physicians. Based on previous validated tool and a pilot tested tool. Theory of planned behaviour successfully predicted physician behaviours. 84-item instrument about characteristics, knowledge, attitudes and intention to administer regarding the HPV Vaccine. MANOVA performed to examination association between intention and patient age, patient gender, and vaccine type. Univariate analysis for associations between predictor variables and intention. | (Recommend the HPV vaccine for Cervical cancer by gender and age group) Females 11: 89, Males 11: 57, Females 14: 129, Males 14: 79, Females 17: 137, Males 17: 92 | Reluctance to discuss sexuality and time pressure. Most frequently reported barriers were related to parental barriers to vaccination. |
| **Roland, et al, 2014** | USA | Present recommendations and beliefs of providers in the Cervical Cancer study. | Physicians, nurse practitioners, CNMs and PAS. | All providers who routinely provide cervical cancer screenings were eligible. HPV recommendation assessed by asking, to what age groups do you recommend the HPV vaccine, how often do you recommend to women with abnormal pap smears, to women with positive HPV test, use HPV test to determine who should get the vaccine, or preform a pap smear to determine who should get the vaccine, or number of sexual partner. Will your cervical cancer screening procedures change for those who are fully vaccinated. | 93% of providers currently recommend or planned to recommend HPV to their patients. Most commonly for 13-26 year olds, females between 9-12, males 12-26. | N/A |
| **Selvan, et. al, 2021** | USA | The aim of the study was to assess the knowledge, attitudes, and practices of nurses in an urban school district regarding human papillomavirus (HPV) vaccination. | Nurses | It is a cross-sectional study design with an 18-item survey about nurses' knowledge, attitudes, and role in promoting the HPV vaccine. | 65 school nurses (31%) agreed or strongly agreed with the statement ‘‘if I could provide the vaccine to the students at my school(s), I would,’’ | The barriers to HPV vaccination include concern about vaccine safety and side effects, lack of a physician recommendation, mistrust in the health care system, particularly pharmaceutical companies, lack of knowledge of cervical cancer HPV and the vaccine, and cost. |
| **Sherman, et. al, 2018** | New Zealand | The aim of the study was to assess the knowledge, attitudes, and awareness of health professionals in New Zealand regarding the human papillomavirus (HPV). The study aimed to identify any gaps in knowledge and determine the level of confidence among health professionals in answering HPV-related questions from patients. | Healthcare professionals | The study used a cross-sectional survey method to collect data. The survey had four main areas: general HPV knowledge, HPV Triage and Test of Cure (TOC) knowledge, HPV vaccine knowledge, Self-perceived adequacy of HPV knowledge. | Of all respondents, 96.5% (N = 220) agreed or strongly agreed that they would recommend the HPV vaccine | The study mentions lack of training as a barrier to HPV vaccination recommendations. |
| **Sherman, et al, 2019** | UK | The study aims to assess the knowledge and attitudes of UK HCPs towards HPV, the HPV vaccine, and changes in the screening program. | Health professionals (physicians, nurses, and other clinical and laboratory staff) | The method used in the study was an anonymous cross-sectional survey. The outcome measured in the study is the level of knowledge and attitudes towards HPV, the HPV vaccine, changes to the screening program, and health sequelae for males among UK healthcare professionals (HCPs). | Of all respondents, 98.3% (n= 632) agreed or strongly agreed that they would recommend the HPV vaccine, whereas 88.2% (n= 567) respondents agreed or strongly agreed that men/boys should be offered the vaccine | Half cited a lack of knowledge, 2 questioned its effectiveness against only HPV 16 and 18, 2 questioned why boys aren't vaccinated, and 1 indicated it hasn't been used long enough. |
| **Shuto, et. al, 2021** | Japan | The aim of the study is to understand the level of confidence in the human papillomavirus (HPV) vaccine among mothers, female adolescents, and healthcare professionals in Japan. | Mothers, female adolescent, and Healthcare professionals | This is a cross-sectional, web-based survey of mothers of HPV vaccination–eligible girls, female adolescents, and HCPs in Japan. Understanding HPV vaccine confidence and the willingness to receive the HPV vaccine among Japanese mothers and female adolescents, and among healthcare professionals (HCPs) to recommend the HPV vaccine. | At the time of the survey, 40% of HCPs recommended HPV vaccination to their female adolescent patients. | “Safety concerns” and “insufficient information” were the main drivers among the stakeholders for vaccine refusal or lack of recommendation. |
| **Signorelli, et. al, 2014** | Italy | The aim of the study was to assess the knowledge and attitudes of Italian general practitioners (GPs) about HPV infection and prevention. | General Practitioners and Physician specialists | The study design is a semi-structured survey conducted Using a literature review and expert opinions a questionnaire was developed with three sections: sociodemographic characteristics, degree of knowledge, opinions and attitudes on the vaccine and HPV. | 96% declare themselves in favour of vaccination in 12-year-olds. | The information regarInsuffienct information, poor perception of benefits, refusal by parents. Too recent, and doubts of safety/tolerance. 45% believe it may induce a false send of protection agaist STDs. |
| **Song, et. al 2023** | China | The aim of the study was to explore the knowledge and attitudes towards the human papillomavirus vaccine (HPV) among healthcare providers (HCPs) involved in the governmental free HPV vaccination program in Shenzhen, Southern China. | Healthcare professionals | The study design is a cross-sectional study. The researchers used a convenience sample strategy to distribute questionnaires to healthcare providers involved in the government's HPV vaccination program in Shenzhen, southern China. The outcome variable in the study is the knowledge and attitudes towards HPV vaccination | Out of 770 participants, a total of 729 (94.7%) reported recommending the HPV vaccine to others. | Themain three reasons for this included: “Vaccine promotion is not my responsibility,” “Fear oftrouble caused by recommending self-pay vaccines to service recipients” and “Uncertaintyof the HPV vaccination process” |
| **Soon, et. al, 2015** | USA | The aim of the study was to gather detailed information about Hawaiʻi physicians' practices and attitudes toward HPV vaccination, with a particular focus on whether they recommend and/or administer the vaccine in their offices. | Paediatricians and Family medicine physicians | The study conducted was a cross-sectional survey of practicing physicians specialising in paediatrics or family medicine in Hawaii. The outcome measure of the study is to gather detailed information about physicians' practices and attitudes toward HPV vaccination, with a particular focus on whether they recommend and/or administer the vaccine in their offices. | 71% (50/70) of the physicians reported they strongly recommend the HPV vaccine for girls 11-12 years old, with minimal difference between the paediatricians and family physicians. For boys age 11-12, only 57% (39/68) of physicians strongly recommend the HPV vaccine; 64% (23/36) of paediatricians and 50% (16/32) of family physicians | The study mentions that barriers to providing HPV vaccination include financial reasons such as lack of insurance coverage and the cost of the vaccine. |
| **Staras, et al., 2023** | USA | examined HPV vaccine recommendation practices among Florida clinicians by assessing variability in: (1) recommendation priorities by patient characteristics and (2) concordance with best practices. | primary care clinicians (MD/DO, APRN, and PA) | In 2018 and 2019, we conducted a cross-sectional survey incorporating a discrete choice experiment among primary care clinicians (MD/DO, APRN, and PA). linear mixed-effects models was used to determine the importance of patient characteristics (age, sex, time in practice, and chronic condition) and parental concerns. We compared clinician endorsement of predetermined constructs with reported vaccine recommendation statements. | HPV vaccination recommendation strategies among Florida clinicians somewhat aligned with best practices. Alignment was higher when clinicians were explicitly asked to endorse constructs versus provide recommendations. | N/A |
| **Stamenkovic, et al, 2017** | Serbia | To assess the level of their knowledge and attitudes toward HPV infection and factors associated with the recommendation of HPV vaccine. | All gynaecologists working at women's health departments in all primary health centres in Belgrade, the capital of Serbia. | The study methods involved conducting a survey using a specially designed questionnaire among all gynaecologists working at women's health departments in primary health centres in Belgrade, Serbia. The research was carried out between April and June 2014 to assess gynaecologists’ knowledge, attitudes, and intentions toward human papillomavirus (HPV) infection and the HPV vaccine. | More than two thirds of the healthcare providers, specifically 68.4%, were willing to recommend the HPV vaccine. | Barriers to HPV vaccine recommendation by healthcare providers include financial concerns, which was the most frequently reported obstacle at 59.8%. |
| **Steben et al, 2019** | Canada | This survey of Canadian physicians aimed to explore knowledge, barriers, and preventive practices regarding HPV vaccination | General practitioners (GPs) andobstetrician/gynaecologists. These physicians were surveyed to gather insights into their knowledge, barriers, and preventive practices concerning HPV vaccination. | The study utilized a survey methodology to collect data from Canadian physicians. The physicians were asked questions related to their knowledge, barriers, and preventive practices regarding HPV vaccination. The survey responses were then analysed to generate the findings mentioned in the summary. To obtain these findings, demographic information about the physicians, such as their gender, were collected and analysed as well. | A total of 83% of general practitioners (GPs) recommended or administered the HPV vaccine to adults | The study revealed that a substantial percentage of physicians identified cost as the primary barrier, and concerns about consumers' limited understanding of HPV as influencing vaccination recommendations. |
| **Sypien, et al. 2023** | Poland | The aim of the study was to evaluate gynaecologists’ and general practitioners’ knowledge, awareness and attitudes towards HPV vaccination and analyse their opinions about the interest in HPV vaccinations among children and parents. | Medical practitioners including gynaecologists and general practitioners specializing in paediatric care. | The study utilized a questionnaire with 29 questions across three sections: demographic information, doctors' attitudes and experiences regarding HPV vaccinations, doctors' knowledge about HPV vaccinations through five specific questions. Statistical analysis presenting data as frequencies and percentages, with the chi-square test used for categorical variable comparisons (p<0.05 for significance). | Around 83% of respondents reported providing information about the HPV-related diseases and vaccines to young patients and their parents. | Barriers to HPV vaccine recommendation by healthcare providers include financial concerns, uncertainty about vaccine effectiveness, concerns about side effects, a small percentage of doctors opposing vaccinations, and varied emphasis on HPV risks by different types of healthcare provider. |
| **Thaker, et al., 2023** | USA | To determine nurses’ perceptions, experiences, and practices regarding human papillomavirus vaccination in a rural and medically underserved region of the United States. | nurses and medical assistants | Designed, pilot-tested, and disseminated an online survey instrument to nurses and medical assistants working in clinics participating in the Vaccines for Children program in Montana. Survey questions focused on clinic vaccination practices, respondents’ perceptions of the HPV vaccine, perceived barriers to vaccine uptake, and general opinions on potential strategies to improve HPV vaccination rates. | Likelihood of Recommending is above average | Misinformation from social media, infrequent wellness visits, and vaccine safety concerns |
| **Tron, et al., 2023** | France | To determine the perspective and behaviours of general practitioners (GPs) regarding HPV vaccination with their patients and if a reluctance is observed. | GP | A qualitative study based on semi-directed individual interviews was conducted between December 2019 and December 2020. A representative sample of GPs with various profiles were included in 4 French regions. A purposive sampling was used, and interviews were continued until data saturation was reached. The analysis was based on the grounded theory | Different practices were identified according to three GP typologies: effective, convinced but unpersuasive, and reluctant physicians. | barriers were organizational, due to low attendance of adolescents, and relational, mainly due to parental vaccine hesitancy. |
| **Tolentin et al, 2018** | USA | The study aimed to assess the knowledge, attitudes, and barriers of Utah community pharmacists regarding the recommendation of the human papillomavirus (HPV) vaccine. | Community pharmacists. | The study surveyed 240 Utah community pharmacists to assess their knowledge, attitudes, and barriers regarding recommending the HPV vaccine. The survey results were analysed to understand pharmacists' perspectives on the vaccine and their recommendation behaviours. | The study found that only one-third of pharmacists recommended the HPV vaccine for both boys and girls. | The top 3 reported barriers included lack of parental knowledge, parental concerns and opposition, and lack of educational materials to provide parents. |
| **Tolunay,et al, 2014** | Turkey | The aim of the study was to determine the level of knowledge on human papillomavirus (HPV) infection and vaccination, as well as the attitude towards HPV vaccination among paediatricians, obstetricians, and gynaecologists in Turkey. | Paediatricians and OB/GYNs | A total of 228 participants, including 131 paediatricians and 97 obstetricians/gynaecologists, were surveyed using a 40-question survey. The survey covered demographic information, knowledge about HPV infection and vaccination, and attitudes towards vaccination. Statistical analysis was conducted using the "Statistical Package for Social Sciences" software. | Among the participants, 59.4% of paediatric specialists recommended the HPV vaccine, while 82.6% of obstetrics and gynaecology specialists, 66.7% of obstetrics and gynaecology residents, and 80.6% of paediatric residents recommended the vaccine. Additionally, 79.5% of the participants expressed a favourable attitude towards having their daughters receive the HPV vaccine. However, the rate was lower for sons, with only 36.7% of participants indicating a favorable response. | Reasons for not recommending include lack of efficacy of the vaccine, the high cost of the vaccine, the side effects of the vaccine and other causes. |
| **Wong, et al. 2016** | Malaysia | The aim of the study was to assess physicians' experiences in recommending and delivering the HPV vaccine to adolescent boys in Malaysia. The study sought to understand the issues surrounding vaccine acceptance among adolescent boys and provide insights for policymakers to improve HPV vaccination rates in this specific population. | Physicians | The total sample size for the study was 357. | Only 26.3% (n = 88) of healthcare workers recommended the HPV vaccine to boys. | A lack of proper guidelines from the health authorities regarding the recommendation of HPV vaccine to boys and a lack of awareness of the availability of the vaccine for boys were the most commonly cited reasons for non-recommendation. |
| **Wong, MC et al. 2013** | Hong Kong | To evaluate the knowledge on HPV infection, and the attitude towards and perceived barriers of HPV vaccination among Primary Care Physicians in Hong Kong. Their practice of prescribing HPV vaccine according to recommendations and patient characteristics was also explored. | Primary Care Physicians | This is a cross-sectional study among Primary Care Physicians in Hong Kong. The study used a self-administered questionnaire survey to assess their knowledge, attitude, practice, and barriers regarding HPV vaccination. The survey was sent to doctors who had participated in a vaccination program for school girls. The survey instrument used a Likert scale for assessing various aspects related to HPV vaccination among primary care physicians in Hong Kong. | A substantial proportion of physicians had recommended HPV vaccines for their female clients/patients aged 18–26 years for protection of cervical cancer (83.8%) or both cervical cancer and genital warts (85.5%). | The most significant barriers to prescribe HPV vaccines consisted of parental refusal due to safety concerns (48.2%), and their practice of advising vaccination was mostly affected by local Governmental recommendations (78.7%). |
| **Xu, et al., 2021** | China | To assess healthcare providers' knowledge, attitudes, and recommendations regarding HPV vaccines. | Healthcare providers including those from the Division of Expanded Program on Immunization, Community Health Centres, obstetrician-gynaecologists, and other non-HPV related professions. | The 55-question questionnaire covered socio-demographic characteristics, awareness of HPV and its vaccines, knowledge levels, willingness to recommend vaccination, and opinions on appropriate target populations. Data collection occurred between April 2019 and October 2019 through an electronic survey tool, with respondents categorized into professional groups, including those from the Division of Expanded Program on Immunization, Community Health Centres, obstetrician-gynaecologists, and other healthcare providers. | About 74.8% of healthcare providers in China reported recommending HPV vaccination | Barriers to HPV vaccine recommendation among healthcare providers in China, including knowledge gaps related to risk factors of HPV infection, the best time to vaccinate, and the classification of low-risk and high-risk types of HPV. |
| **Yetik, et al. 2023** | Turkey | to evaluate the level of knowledge, compliance with the screening program, and tendency to inform patients of the doctors working in FHCs where HPV testing is performed within the scope of the cervical cancer screening program in Turkey. | Family Physician | This cross-sectional study was performed between June and September 2022 with 113 family physicians working in different FHCs in different provinces in Turkey. Questionnaires prepared by the researchers were delivered to family physicians via online platforms. | 105 (92.9%) recommended the HPV vaccine to patients and their relatives and 60 (53.1%) recommended the HPV vaccine to patients who applied for another reason. | N/A |
| **Yu, et. al, 2020** | China | The aim of the study was to investigate the knowledge about HPV vaccines and willingness to accept or recommend HPV vaccination among medical staff, medical students, and community members in Fujian Province. | Medical staff, medical students, and community members | The study design used in this research is a cross-sectional survey. The study outcome measured in this study were HPV knowledge, intention to receive the HPV vaccine, and intention to recommend HPV vaccination. | In our survey, 63.7% of medical staff indicated their willingness to recommend vaccines | The reasons determining the willingness to vaccinate were different between the three groups, with medical staff selecting doubts on the source and Vaccination causes risks; medical students choose Vaccines are too expensive; community members choose not yet widely used. |
| **Yuta, et al, 2023** | Japan | his study aimed to evaluate the association between physicians’ knowledge of vaccination and the administration or recommendation of HPVv by primary care physicians (PCPs) in the absence of proactive recommendations from the Japanese government. | Primary care physicians (PCPs) | Cross-sectional study analysed data obtained through a web-based, self-administered questionnaire survey. The questionnaire was distributed to Japan Primary Care Association (JPCA) members’ members who were physicians and on the official JPCA mailing list (n=5395) were included. | The PCPs selected the following options regarding the recommendation of HPVv under routine vaccination: ‘actively recommend’, 408 PCPs (41.6%); ‘recommend occasionally’, 319 PCPs (32.5%); ‘no opinion’, 181 PCPs (18.5%); ‘do not actively recommend’, 49 (5.0%) and ‘do not recommend’, 24 (2.5%) (table 2). PCP with higher scores recommended the HPV vaccine for routine and voluntary vaccination than PCPs with a lower score | N/A |
| **Zhang, et al., 2023** | USA | To assess the impact of an online, asynchronous educational module on HPV vaccination for adult primary care providers | Primary care providers | We designed and implemented the module for family medicine, internal medicine, medicine/paediatrics, and obstetrics/gynaecology providers in a community practice network affiliated with a large academic health system. We evaluated the effect of the module on provider knowledge, attitudes, and self-reported behaviours with pre-, post-, and delayed post-tests, using Likert-scales for measurement. We summarized data with descriptive statistics and compared changes in individuals using paired t-tests. | Participants were more likely to recommend HPV vaccination for eligible patients as a result of online course, internal medicine providers were the least likely to discuss HPV vaccination with their patients. OB/GYN providers were the most likely to discuss HPV vaccination with their patients. | N/A |
