# Supplementary material for: Healthcare worker practices for HPV vaccine recommendation: A systematic review and meta-analysis
Source: Hum Vaccin Immunother. 2024 Oct 14;20(1):2402122. doi: 10.1080/21645515.2024.2402122 (PMC11486212; doi:10.1080/21645515.2024.2402122)
Supplement: Appendix 2_Documentation of search strategies.docx [file KHVI_A_2402122_SM6232.docx]

Documentation of search strategies

University Library search consultation group

Date: Update March 2024

Topic/research question: What survey tools/instruments have been tested and used to assess health worker vaccine confidence?

Name of researcher(s): Sibylle Herzig van Wees & Elisa Gobbo, Global Public Health

Librarian(s): Narcisa Hannerz & peer-reviewed by Sabina Gillsund

Databases:

1. Medline (Ovid)
2. Web of Science (Clarivate Analytics)
3. CABI: CAB Abstracts & Global Health (Clarivate Analytics) - **not available during update 2024**
4. Sociological Abstracts (ProQuest)
5. Publicly Available Content Database‎ (ProQuest)

Total number of hits:

- Before deduplication: 20,238 (fererences from Global Health from 2023)
- After deduplication: 10,877

Text that can be used in the Methods-section:

Search strategy

A literature search was performed in the following databases: Medline, Web of Science, CABI: CAB Abstracts and Global Health and Sociological Abstracts and as a complementary search Publicly Available Content database was used. In CABI the last search was conducted 2023-06-08, after the original search, the search was last updated in the other database on 2024-03-19 using the methods described by Bramer et al (1). During that time access to CABI was not available.

The search strategy was developed in Medline (Ovid) in collaboration with librarians at the Karolinska Institutet University Library. For each search concept Medical Subject Headings (MeSH-terms) and free text terms were identified. The search was then translated, in part using Polyglot Search Translator (2), into the other databases.

No language restriction was applied and databases were searched from inception.

The strategies were peer reviewed by another librarian prior to execution.

De-duplication was done using the method described by Bramer et al (3). One final, extra step was added to compare DOIs.

The full search strategies for all databases are available in the appendix.

References

1. Bramer W, Bain P. (2017). Updating search strategies for systematic reviews using EndNote. *Journal of the Medical Library Association: JMLA*, 105(3):285-289. doi: 10.5195/jmla.2017.183.

2. Clark JM, Sanders S, Carter M, Honeyman D, Cleo G, Auld Y, Booth D, Condron P, Dalais C, Bateup S, Linthwaite B, May N, Munn J, Ramsay L, Rickett K, Rutter C, Smith A, Sondergeld P, Wallin M, Jones M, Beller E. (2020) Improving the translation of search strategies using the Polyglot Search Translator: a randomized controlled trial. *Journal of the Medical Library Association: JMLA*. 108(2):195-207. doi: 10.5195/jmla.2020.834.

3. Bramer, W. M., Giustini, D., de Jonge, G. B., Holland, L., & Bekhuis, T. (2016). De-duplication of database search results for systematic reviews in EndNote. *Journal of the Medical Library Association: JMLA*, 104(3), 240-243. doi: 10.3163/1536-5050.104.3.014

Records removed *before screening*:

Duplicate records removed (n = 9,361)

Records marked as ineligible by automation tools (n = )

Records removed for other reasons (n = )

Records identified from*:

Databases (n = 20,238

Medline = 7,615

Web of Science = 5,136

CABI: CAB Abstracts and Global Health = 4,702

Sociological Abstracts = 297

Publicly Available Content Database = 2,488)

Registers (n = )

Reports assessed for eligibility

(n = )

**Identification of studies via databases and registers**

**Identification of studies via other methods**

Records screened

(n = 10,877)

Records excluded**

(n = )

Reports sought for retrieval

(n = )

Reports sought for retrieval

(n = )

Reports not retrieved

(n = )

**Screening**

Reports excluded:

Reason 1 (n = )

Reason 2 (n = )

Reason 3 (n = )

etc.

Reports assessed for eligibility

(n = )

Reports excluded:

Reason 1 (n = )

Reason 2 (n = )

Reason 3 (n = )

etc.

Records identified from:

Websites (n = )

Organisations (n = )

Citation searching (n = )

etc.

**Identification**

Reports not retrieved

(n = )

Studies included in review

(n = )

Reports of included studies

(n = )

**Included**

*Consider, if feasible to do so, reporting the number of records identified from each database or register searched (rather than the total number across all databases/registers).

**If automation tools were used, indicate how many records were excluded by a human and how many were excluded by automation tools.

*From:*  Page MJ, McKenzie JE, Bossuyt PM, Boutron I, Hoffmann TC, Mulrow CD, et al. The PRISMA 2020 statement: an updated guideline for reporting systematic reviews. BMJ 2021;372:n71. doi: 10.1136/bmj.n71. For more information, visit: <http://www.prisma-statement.org/>

1. Medline

| Interface: Ovid MEDLINE(R) ALL  Date of Search: 19 March 2024  Number of hits: 7,615  Comment: In Ovid, two or more words are automatically searched as phrases; i.e. no quotation marks are needed | Field labels   - exp/ = exploded MeSH term - / = non exploded MeSH term - .ti,ab,kf. = title, abstract and author keywords - adjx = within x words, regardless of order - * = truncation of word for alternate endings |
| --- | --- |
| Database(s): **Ovid MEDLINE(R) ALL**1946 to March 18, 2024 Search Strategy:   \| **#** \| **Searches** \| **Results** \| \| --- \| --- \| --- \| \| 1 \| Immunization/ \| 54093 \| \| 2 \| Immunization Programs/ \| 13019 \| \| 3 \| exp Vaccination/ \| 113500 \| \| 4 \| exp Vaccines/ \| 285527 \| \| 5 \| (immuni?at* or nonvaccin* or non-immun* or nonimmun* or unimmun* or un-immun* or unvaccin* or vaccin*).ti,ab,kf. \| 515238 \| \| 6 \| or/1-5 \| 591985 \| \| 7 \| Anti-Vaccination Movement/ \| 182 \| \| 8 \| Patient Acceptance of Health Care/ \| 55630 \| \| 9 \| exp Vaccination Refusal/ \| 1854 \| \| 10 \| (anti-vaccin* or antivaccin* or anti-vax* or antivax*).ti,ab,kf. \| 1327 \| \| 11 \| ((vaccin* or immuni?at*) adj3 (confiden* or delay* or hesitan* or refuse? or refusing or refusal*)).ti,ab,kf. \| 11077 \| \| 12 \| Anxiety/ \| 111555 \| \| 13 \| Awareness/ \| 22238 \| \| 14 \| Behavior/ \| 30208 \| \| 15 \| Choice Behavior/ \| 35146 \| \| 16 \| Communication Barriers/ \| 7354 \| \| 17 \| Consciousness/ \| 13999 \| \| 18 \| Decision Making/ \| 105712 \| \| 19 \| Fear/ \| 39700 \| \| 20 \| Health Knowledge, Attitudes, Practice/ \| 128027 \| \| 21 \| Intention/ \| 17279 \| \| 22 \| exp Mandatory Programs/ \| 7216 \| \| 23 \| Trust/ \| 13517 \| \| 24 \| ((immuni?at* or vaccin*) adj3 (accept* or anxi* or attitude* or awareness or barrier* or behavio?r* or belief* or choice* or compulsory or concern* or conscious* or controvers* or critic* or decision-make* or decision-making* or dilemma* or distrust or doubt* or dropout* or enable* or exemption* or fear* or intent* or knowledge or mandatory or misconception* or misinformat* or mistrust* or objection* or objector* or opposition* or perception* or reject* or reluctan* or rumo?r* or trust* or uptake* or willing* or unconscious* or unwilling*)).ti,ab,kf. \| 31378 \| \| 25 \| exp Vaccination/px \| 2876 \| \| 26 \| or/7-25 \| 568360 \| \| 27 \| 6 and 26 \| 42596 \| \| 28 \| exp Health Personnel/ \| 629172 \| \| 29 \| ((clinical or health or health care or healthcare or medical) adj3 (personnel or professional* or provider* or staff or worker*)).ti,ab,kf. \| 392558 \| \| 30 \| (clinician* or general practitioner* or nurse* or pharmacist* or physician*).ti,ab,kf. \| 1135896 \| \| 31 \| or/28-30 \| 1760982 \| \| 32 \| 27 and 31 \| 11792 \| \| 33 \| Benchmarking/ \| 18741 \| \| 34 \| Health Care Surveys/ \| 34072 \| \| 35 \| Quality Assurance, Health Care/ \| 56948 \| \| 36 \| "Surveys and Questionnaires"/ \| 575605 \| \| 37 \| (benchmark* or best practice analy* or feedback* or form? or instrument? or metric? or measure* or nonrespondent? or non-respondent? or questionnair* or respondent* or survey* or tool or tools).ti,ab,kf. \| 7549215 \| \| 38 \| ((assessment* or assurance or qualit*) adj3 (care or health care or healthcare)).ti,ab,kf. \| 134363 \| \| 39 \| or/33-38 \| 7770261 \| \| 40 \| 32 and 39 \| 7615 \| | |

2. Web of Science Core Collection

| Interface: Clarivate Analytics  Editions = A&HCI , ESCI , SCI-EXPANDED , SSCI  Date of Search: 19 March 2024  Number of hits: 5,136 | Field labels   - TS/Topic = title, abstract, author keywords and Keywords Plus - NEAR/x = within x words, regardless of order - * = truncation of word for alternate endings   Note: the *Exact search*-function was used for all the searches |
| --- | --- |
| \| # \| Search Query \| Results \| \| --- \| --- \| --- \| \| 1 \| TS=(immuni$at* OR nonvaccin* OR non-immun* OR nonimmun* OR unimmun* OR un-immun* OR unvaccin* OR vaccin* ) \| 543942 \| \| 2 \| TS=(anti-vaccin* OR antivaccin* OR anti-vax* OR antivax* ) \| 1539 \| \| 3 \| TS=((vaccin* OR immuni$at* ) NEAR/2 (confiden* OR delay* OR hesitan* OR refuse$ OR refusing OR refusal* )) \| 11273 \| \| 4 \| TS=((immuni$at* OR vaccin* ) NEAR/2 (accept* OR anxi* OR attitude* OR awareness OR barrier* OR behavio$r* OR belief* OR choice* OR compulsory OR concern* OR conscious* OR controvers* OR critic* OR decision-make* OR decision-making* OR dilemma* OR distrust OR doubt* OR dropout* OR enable* OR exemption* OR fear* OR intent* OR knowledge OR mandatory OR misconception* OR misinformat* OR mistrust* OR objection* OR objector* OR opposition* OR perception* OR reject* OR reluctan* OR rumo$r* OR trust* OR uptake* OR willing* OR unconscious* OR unwilling* )) \| 30819 \| \| 5 \| #2 OR #3 OR #4 \| 35891 \| \| 6 \| TS=((clinical OR health OR "health care" OR healthcare OR medical ) NEAR/3 (personnel OR professional* OR staff OR worker* )) \| 287188 \| \| 7 \| TS=(clinician* OR "general practitioner*" OR nurse* OR pharmacist* OR physician* ) \| 952723 \| \| 8 \| #6 OR #7 \| 1163626 \| \| 9 \| TS=(benchmark* OR "best practice analy*" OR feedback* OR form$ OR instrument$ OR metric$ OR measure* OR nonrespondent$ OR non-respondent$ OR questionnair* OR respondent* OR survey* OR tool OR tools ) \| 13377734 \| \| 10 \| TS=((assessment* OR assurance OR qualit* ) NEAR/2 (care OR "health care" OR healthcare )) \| 125298 \| \| 11 \| #9 OR #10 \| 13438191 \| \| 12 \| #1 AND #5 AND #8 AND #11 \| 5136 \| | |

3. CABI: CAB Abstracts and Global Health

| Interface: Clarivate Analytics  Date of Search: **8 June 2023**  Number of hits: **4,702** | Field labels   - DE = descriptors - TS/Topic = Abstract, BHTD Critical Abstract, Broad Descriptors, CABICODES Names, Descriptors, English Title, Foreign Title, Geographic Location, Identifiers, Organism Descriptors - NEAR/x = within x words, regardless of order - * = truncation of word for alternate endings   Note: the *Exact search*-function was used for all the searches |
| --- | --- |
| \| # \| Search Query \| Results \| \| --- \| --- \| --- \| \| 1 \| TS= (immuni$at* OR nonvaccin* OR non-immun* OR nonimmun* OR unimmun* OR un-immun* OR unvaccin* OR vaccin* ) \| 348,475 \| \| 2 \| DE = (vaccines OR DNA vaccines OR Haemophilus influenzae vaccines OR acellular vaccines OR autogenous vaccines OR candidate vaccines OR cell culture vaccines OR combined vaccines OR conjugate vaccines OR inactivated vaccines OR live vaccines OR malaria vaccines OR pertussis vaccines OR poliomyelitis vaccines OR polyvalent vaccines OR recombinant vaccines OR synthetic vaccines OR whole cell vaccines) \| 172,519 \| \| 3 \| DE = (immunization programmes) OR DE = (vaccination OR mandatory vaccination OR oral vaccination) \| 144,164 \| \| 4 \| DE = (immunization) \| 145,047 \| \| 5 \| #1 OR #2 OR #3 OR #4 \| 348,475 \| \| 6 \| DE = (anxiety) \| 21,782 \| \| 7 \| DE = (awareness) \| 15,771 \| \| 8 \| DE = (behaviour) \| 321,707 \| \| 9 \| DE = (social barriers) \| 1,333 \| \| 10 \| DE = (consciousness OR social consciousness) \| 1,018 \| \| 11 \| DE = (decision making) \| 55,532 \| \| 12 \| DE = (knowledge OR attitudes OR practice) \| 209,545 \| \| 13 \| TS=((immuni$at* OR vaccin* ) NEAR/2 (accept* OR anxi* OR attitude* OR awareness OR barrier* OR behavio$r* OR belief* OR choice* OR compulsory OR concern* OR conscious* OR controvers* OR critic* OR decision-make* OR decision-making* OR dilemma* OR distrust OR doubt* OR dropout* OR enable* OR exemption* OR fear* OR intent* OR knowledge OR mandatory OR misconception* OR misinformat* OR mistrust* OR objection* OR objector* OR opposition* OR perception* OR reject* OR reluctan* OR rumo$r* OR trust* OR uptake* OR willing* OR unconscious* OR unwilling* )) \| 17,343 \| \| 14 \| TS=(anti-vaccin* OR antivaccin* OR anti-vax* OR antivax* ) \| 658 \| \| 15 \| TS=((vaccin* OR immuni$at* ) NEAR/2 (confiden* OR delay* OR hesitan* OR refuse$ OR refusing OR refusal* )) \| 5,055 \| \| 16 \| #6 OR #7 OR #8 OR #9 OR #10 OR #11 OR #12 OR #13 OR #14 OR #15 \| 571,642 \| \| 17 \| DE = (health workers OR health care workers OR careproviders OR community health workers OR dentists OR dietitians OR home health aides OR midwives OR nurses OR nutritionists OR physicians OR traditional birth attendants OR traditional healers) \| 97,699 \| \| 18 \| TS=((clinical OR health OR "health care" OR healthcare OR medical ) NEAR/2 (personnel OR professional* OR provider* OR staff OR worker* )) \| 113,305 \| \| 19 \| TS=(clinician* OR "general practitioner*" OR nurse* OR pharmacist* OR physician* ) \| 224,720 \| \| 20 \| #17 OR #18 OR #19 \| 323,578 \| \| 21 \| #5 AND #16 AND #20 \| 6,910 \| \| 22 \| DE = (quality assurance) \| 0 \| \| 23 \| DE = (questionnaires) \| 52,923 \| \| 24 \| DE = (surveys) \| 247,268 \| \| 25 \| TS=(benchmark* OR "best practice analy*" OR feedback* OR form$ OR instrument$ OR metric$ OR measure* OR nonrespondent$ OR non-respondent$ OR questionnair* OR respondent* OR survey* OR tool OR tools ) \| 3,551,736 \| \| 26 \| TS=((assessment* OR assurance OR qualit* ) NEAR/2 (care OR "health care" OR healthcare )) \| 24,641 \| \| 27 \| #22 OR #23 OR #24 OR #25 OR #26 \| 3,563,318 \| \| 28 \| #27 AND #21 \| 4,719 \| \| 29 \| #28 AND CABI: Global Health (CABI Index) \| 4,702 \| | |

4. Sociological abstracts

| Interface: ProQuest  Date of Search: 19 March 2024  Number of hits: 297  Comment: Includes companion file Social Services Abstracts | Field labels   - noft = anywhere except full text - tiabif = title, abstract, keyword - MAINSUBJECT.EXACT = non exploded subject heading - MAINSUBJECT.EXACT.EXPLODE = exploded subject heading - NEAR/x = within x words, regardless of order - * = truncation of word for alternate endings   Note: sometimes “quotation marks” are needed for single search terms to avoid automatic term mapping (lemmatization). |
| --- | --- |
| \| S1 \| MAINSUBJECT.EXACT("Vaccination") OR (TI,AB,IF(immunizat* OR immunisat OR nonvaccin* OR non-immun* OR nonimmun* OR unimmun* OR un-immun* OR unvaccin* OR vaccin*)) \| 3,537 \| \| --- \| --- \| --- \| \| S2 \| MAINSUBJECT.EXACT("Anxiety") OR MAINSUBJECT.EXACT("Awareness") OR MAINSUBJECT.EXACT("Consciousness") OR MAINSUBJECT.EXACT("Choices") OR (MAINSUBJECT.EXACT("Medical Decision Making") OR MAINSUBJECT.EXACT("Decision Making")) OR MAINSUBJECT.EXACT("Fear") OR MAINSUBJECT.EXACT("Intentionality") OR MAINSUBJECT.EXACT("Trust") OR ((immunizat* OR immunisat* OR vaccin*) NEAR/3 (confiden* OR delay* OR hesitan* OR refuse OR refuses OR refusing OR refusal* )) OR (TI,AB,IF(anti-vaccin* OR antivaccin* OR anti-vax* OR antivax* )) OR ((immunizat* OR immunisat* OR vaccin* ) NEAR/3 (accept* OR anxi* OR attitude* OR awareness OR barrier* OR behavior* OR behaviour* OR belief* OR choice* OR compulsory OR concern* OR conscious* OR controvers* OR critic* OR decision-make* OR decision-making* OR dilemma* OR distrust OR doubt* OR dropout* OR enable* OR exemption* OR fear* OR intent* OR knowledge OR mandatory OR misconception* OR misinformat* OR mistrust* OR objection* OR objector* OR opposition* OR perception* OR reject* OR reluctan* OR rumor* OR rumour* OR trust* OR uptake* OR willing* OR unconscious* OR unwilling* )) \| 78,170 \| \| S3 \| MAINSUBJECT.EXACT.EXPLODE("Medical personnel") OR ((clinical OR health OR "health care" OR healthcare OR medical) NEAR/3 (personnel OR professional* OR provider* OR staff OR worker*)) OR (TI,AB,IF(clinician* OR ("general practitioner" OR "general practitioners") OR nurse* OR pharmacist* OR physician*)) \| 123,631 \| \| S4 \| MAINSUBJECT.EXACT("Measures") OR MAINSUBJECT.EXACT("Polls & surveys") OR MAINSUBJECT.EXACT("Questionnaires") OR MAINSUBJECT.EXACT("Research Responses") OR (ti,ab,if(benchmark* OR "best practice analy*" OR feedback* OR form OR instrument OR metric OR measure* OR nonrespondent* OR non-responde* OR questionnair* OR responde* OR survey* OR tool OR tools)) OR (ti,ab,if((assessment* OR assurance OR qualit*) NEAR/3 (care OR "health care" OR healthcare))) \| 636,348 \| \| S5 \| [S1] AND [S2] AND [S3] AND [S4] \| 297 \| | |

5. Publicly Available Content Database‎

| Interface: ProQuest  Date of Search: 19 March 2024  Number of hits: 2,488 | Field labels   - noft = anywhere except full text - tiabif = title, abstract, keyword - MAINSUBJECT.EXACT = non exploded subject heading - MAINSUBJECT.EXACT.EXPLODE = exploded subject heading - NEAR/x = within x words, regardless of order - * = truncation of word for alternate endings   Note: sometimes “quotation marks” are needed for single search terms to avoid automatic term mapping (lemmatization). |
| --- | --- |
| \| S1 \| title,abstract(immunisat* OR immunizat* OR nonvaccin* OR non-immun* OR nonimmun* OR unimmun* OR un-immun* OR unvaccin* OR vaccin*) \| 78,706 \| \| --- \| --- \| --- \| \| S2 \| title,abstract(anti-vaccin* OR antivaccin* OR anti-vax* OR antivax*) \| 428 \| \| S3 \| title,abstract((vaccin* OR immuni?at*) NEAR/3 (confiden* OR delay* OR hesitan* OR refuse? OR refusing OR refusal*)) \| 3,319 \| \| S4 \| ((immunizat* OR immunisat* OR vaccin* ) NEAR/3 (accept* OR anxi* OR attitude* OR awareness OR barrier* OR behavior* OR behaviour* OR belief* OR choice* OR compulsory OR concern* OR conscious* OR controvers* OR critic* OR decision-make* OR decision-making* OR dilemma* OR distrust OR doubt* OR dropout* OR enable* OR exemption* OR fear* OR intent* OR knowledge OR mandatory OR misconception* OR misinformat* OR mistrust* OR objection* OR objector* OR opposition* OR perception* OR reject* OR reluctan* OR rumor* OR rumour* OR trust* OR uptake* OR willing* OR unconscious* OR unwilling* )) \| 38,042 \| \| S5 \| [S2] OR [S3] OR [S4] \| 38,502 \| \| S6 \| [S1] AND [S5] \| 25,450 \| \| S7 \| title,abstract((clinical OR health OR "health care" OR healthcare OR medical) NEAR/3 (personnel OR professional* OR provider* OR staff OR worker*)) \| 90,257 \| \| S8 \| title,abstract(clinician* OR ("general practitioner" OR "general practitioners") OR nurse* OR pharmacist* OR physician*) \| 140,498 \| \| S9 \| [S7] OR [S8] \| 209,953 \| \| S10 \| [S6] AND [S9] \| 4,049 \| \| S11 \| title,abstract(benchmark* OR "best practice analy*" OR feedback* OR form? OR instrument? OR metric? OR measure* OR nonrespondent? OR non-respondent? OR questionnair* OR respondent* OR survey* OR tool OR tools) \| 2,720,540 \| \| S12 \| title,abstract((assessment* OR assurance OR qualit*) NEAR/3 (care OR "health care" OR healthcare)) \| 28,373 \| \| S13 \| [S11] OR [S12] \| 2,733,054 \| \| S14 \| [S10] AND [S13] \| 2,488 \| | |
