# Supplementary material for: Healthcare worker practices for HPV vaccine recommendation: A systematic review and meta-analysis
Source: Hum Vaccin Immunother. 2024 Oct 14;20(1):2402122. doi: 10.1080/21645515.2024.2402122 (PMC11486212; doi:10.1080/21645515.2024.2402122)
Supplement: Appendix 1_Healthcare worker and HPV systematic review protocol.docx [file KHVI_A_2402122_SM6231.docx]

**Healthcare worker and HPV systematic review protocol**

**Administrative information**

**Title:** Healthcare workers’ sentiments and behaviours on recommending the HPV Vaccine: a systematic review protocol

**Start Date:** June 2023

**Anticipated completion date:** April 2024

**Registration:** N/A

**Authors**

**Named contact:**

Sibylle Herzig van Wees

[sibylle.hvw@ki.se](mailto:sibylle.hvw@ki.se)

Assistant Professor, PhD, MSc, Global Public Health Department, Karolinska Institute, Stockholm, Sweden

K9 Global folkhälsa, K9 GPH Hanson, 171 77 Stockholm

**Collaborators:**

Elisa Gobbo^a^, Ayobami A. Bakare ^b^, Kofoworola O. Akinsola^c^, Carina King^d,^ Claudia Hanson^e^, Julius Salako^f^, Damola Bakare^g^, Adegoke Falade^h^, Sibylle Herzig van Wees^i^

Affiliations

1. Research Assistant, MSc, Global Public Health Department, Karolinska Institutet, Stockholm Sweden, [elisa.gobbo@ki.se](mailto:elisa.gobbo@ki.se)
2. Senior Registrar, MBBS, MPH, Department of Community Medicine University College Hospital, Ibadan Nigeria; [bakare.ayobami.adebayo@ki.se](mailto:bakare.ayobami.adebayo@ki.se)
3. Research Assistant, MPH, Department of Paediatrics, University College Hospital, Ibadan, Nigeria, [kofoakinsola@gmail.com](mailto:kofoakinsola@gmail.com)
4. Associate Professor, PhD, MSc, Global Public Health Department, Karolinska Institute, Stockholm, Sweden, [carina.king@ki.se](mailto:carina.king@ki.se)
5. Associate Professor, PhD, MSc, [claudia.hanson@ki.se](mailto:claudia.hanson@ki.se)
   1. Global Public Health Department, Karolinska Institute, Stockholm, Sweden,
   2. London School of Hygiene and Tropical Medicine, London, UK
   3. Aga Khan University, Centre of Excellence for Women and Child Health, Nairobi, Kenya
6. Research Assistant, MPH, Department of Paediatrics, University of Ibadan, Ibadan, Nigeria, salakojulius4@gmail.com
7. Research Assistant, MPH, Department of Paediatrics, University of Ibadan, Ibadan, Nigeria, ayodamolabakare@gmail.com
8. Professor and Consultant, MBBS, MD, Paediatrics Department, University College Hospital, Ibadan, Nigeria; Paediatrics Department, University of Ibadan, Ibadan, Nigeria, [afalade33@hotmail.com](mailto:afalade33@hotmail.com)
9. Assistant Professor, PhD, MSc, Global Public Health Department, Karolinska Institute, Stockholm, Sweden, [sibylle.hvw@ki.se](mailto:sibylle.hvw@ki.se)

**Contributions**

SHvW, and AGF conceptualized the project. EG and SHvW will collaborate with the Karolinska Library to formulate the search strategy. EG and KOA will act as the article screeners with team support from AAB and SHvW. EG, KOA, JS, and DB will act as data extractors. CK and DB will conduct the meta-analysis with support from JS, KOA, and EG. Analysis and write up will be supported by CH, AGF, and SWvH. Draft writing is to be done by EG, KOA, and DB. All authors will contribute, read, and approve the final manuscript. SHvW is the guarantor.

**Amendments:**

In January 2024, we then did a rescreening of the initial search with a second researcher to ensure that no articles were missed during the first shorting listing process that fit the inclusion criteria. In this process 26 articles based on title screening that fit eligibility. These were then blinded and rescreened based on the full article screening. This led to 20 new articles included. Conducting the rescreening allowed us to be confident we did not miss any relevant articles.

The search was then rerun in March 2024 with the additional specification for articles relating to HPV vaccine to check for any articles published since the search was completed in June 2023.

**Support**

**Sources:** N/A

**Sponsor/funder:** This project was supported by Vetenskapsrådet (The Swedish Research Council), grant number: 2022-00756. The grant recipient is Sibylle Herzig van Wees.

**Role of funder:**  Veteskapsrådet had no role in conceptualizing the project, developing the protocol, conducting the review, or paper write up.

**Introduction**

**Rationale**

Human papilloma virus (HPV) is linked with 74% of cancers among women, most of which are cervical cancers. The HPV vaccine has been introduced in many countries over the past two decades but roll out is slow globally. Healthcare workers are the most trusted advisor affecting patients or parents’ vaccine decision making. Thus, in an era of increased vaccine hesitancy, understanding healthcare workers vaccine beliefs and recommendation behaviours is critical. The aim of this systematic review is to investigate the evidence that exists on healthcare worker’s sentiments on recommending the HPV vaccine to their patients.

**Objectives**

**Research Question:** What are healthcare workers’ recommendation sentiments and behaviours for the human papillomavirus vaccine?

Rather than using the PICO framework for the research question design, we utilized the population, concept, context (PCC) framework because this systematic review is not accessing intervention studies. For the PCC framework, the population is healthcare workers, the concept is recommendation behaviour, and the context is a global setting.

**Methods**

**Eligibility Criteria:**

The articles will need to focus on vaccine hesitancy/confidence, behaviour, or attitudes. Specifically, the studies need to include data on HPV vaccine recommendation behaviour or sentiments. The inclusion for the population group studied will be HCWs, which includes physicians, nurses, pharmacists, and healthcare administrators. The articles to be included will all be peer review articles. Dentists and students are to be excluded since they were often categorised differently. There will be no language restrictions applied and the databases are to be searched from inception.

**Information Sources:**

**Databases:**

1. Medline (Ovid)
2. Web of Science (Clarivate Analytics)
3. CABI: CAB Abstracts & Global Health (Clarivate Analytics)
4. Sociological Abstracts (ProQuest)
5. Publicly Available Content Database‎ (ProQuest)

**Dates of Coverage:** Inception – June 2023 (search rerun in March 2024)

**Search Strategy:**

For the search strategy, we will begin with a wider search criterion based on the research question; what tools have been used to measure healthcare worker vaccine confidence? Based on this question the full search strategy will be developed with collaboration from the Karolinska Library. Key search terms were immunisation, immunisation programs, exp vaccination, exp vaccines, anxiety, awareness, behaviour, choice behaviour, communication barriers, health knowledge, attitude, and practice, intention, health personnel, benchmarking, health care surveys, quality assurance, health care, survey and questionnaire. (Appendix 2 includes the full search strategy)

**Study Records**

**Data management:** All data will be stored and screened in Rayyan.ai. The software always for maintaining and tracking the articles based on screening inclusion. For extraction processes data management will take place on shared Excel documents.

**Selection process:**

The first round of screening is to be a title screening to include any articles measuring healthcare workers’ vaccine confidence/hesitancy/acceptance. The inclusion criteria during this round will be quite broad and include all healthcare worker types, all vaccines, all study design types, and all measurement strategies. This initial round of screening was done by one researcher and generated 1,717 included articles.

From the breadth of data, we will then narrow to focus on the gap in the literature on a review of healthcare worker sentiments on the HPV vaccine. Thus, we will then conduct blinded abstract and full article screenings based on the research question; what healthcare workers’ recommendation sentiments and behaviours for the human papillomavirus vaccine are.

The 1,717 articles were all blinded and two researchers will do an abstract screening for articles examining healthcare worker sentiments on the HPV vaccine. Then for the final round, to assess the articles for eligibility, the remaining articles will be blinded and screened based on the full eligibility criteria described below. Any discrepancies on the inclusion are to be discussed with the rest of the research team.

**Data collection process:** After the selection process, all of the data will be extracted by four researchers. The data will be split and groups of two researchers each extracted data from half of the included studies. They will extract based on the outcomes listed below for both the narrative collection and the meta-analysis.

**Data items:**

- **General Extraction:**
  - Title
  - Authors
  - Year of Publication
  - Location of Study
  - Background
  - Study Aim
  - Vaccine Type
  - Population Studied
  - Sample Size
  - Methods
  - Outcome Measured
  - Results
  - Barriers
  - Facilitators/Factors associated with Recommending
  - Likelihood to Recommend
- **Meta-Analysis**
  - Author
  - Income level
  - Date of Publication pre or post HPV introduction
  - Study Design
  - Year of Publication
  - Country
  - WHO Region
  - Sample size of recommending
  - Recommendation to boys and girls
  - Willingness to recommend to boys and girls
  - Recommendation to girls
  - Willingness to recommend to girls
  - Recommendation to boys
  - Willingness to recommend to boys

**Outcomes and prioritization:** For the narrative extraction, data on title, authors, year of publication, study location, income level, year of HPV vaccine introduction in study location, background, study aim, vaccination type, study population, sample size, methods, outcome measured, results, facilitators/factors associated with recommending, barriers to recommending, and likelihood of recommending will be gathered. With the associated factors we will look specifically at if studies found a positive association with the recommendation behaviour outcome with female providers, a higher reported knowledge level, urban practice, being an OB/GYN, and being a younger provider. For the meta-analysis, data on sample size, total recommending, total willingness to recommend, total recommending to girls, total willing to recommend to girls, total recommending to boys, and total willing to recommend to boys. The main outcome that we are looking at is the recommendation behaviour or willingness outcome in the studies.

**Risk of Bias:**

We will conduct a quality assessment using JBI assessment tools for cross-sectional and qualitative studies. The studies are to be each assessed blindly by two researchers, and then any discrepancies or clarifications were discussed with the wider team.

**Data synthesis:**

For the data synthesis we will conduct a narrative synthesis of all the articles including those that are qualitative, quantitative, and mixed methods. We will focused on the study population, methods used, conclusions on recommendation sentiments, and barriers in the narrative synthesis. Then we utilized descriptive statistics to summarize the data on year of publication, location, income-level, sample size, and validation process.

We will then conduct a meta-analysis on recommendation practices and willingness to recommend for a subset of articles that gathered quantitative data on either of these outcomes. The data is to be subdivided for those that gathered it for both boys and girls together and those that separated out by gender. For the meta-analyses we will generate using a random effects model in Stata using the metaprop command. We then will also conduct sub-analyses on the studies by study design, pre- or post- HPV introduction during time of publication, income level, and WHO regions.

**Meta-bias:** Not conducted due to the nature of the data as a proportion**.**

**Confidence in cumulative evidence:**

We did not conduct a funnel plot assessment to assess for publication bias because they have been shown to be an inaccurate strategy for assessing proportion studies. As there is no accepted strategy for assessing publication bias for proportions, no measure of confidence in cumulative evidence was undertaken.
