# Supplementary material for: Healthcare worker practices for HPV vaccine recommendation: A systematic review and meta-analysis
Source: Hum Vaccin Immunother. 2024 Oct 14;20(1):2402122. doi: 10.1080/21645515.2024.2402122 (PMC11486212; doi:10.1080/21645515.2024.2402122)
Supplement: Appendix 4_Proportion of HCW recomendation willingness.docx [file KHVI_A_2402122_SM6230.docx]

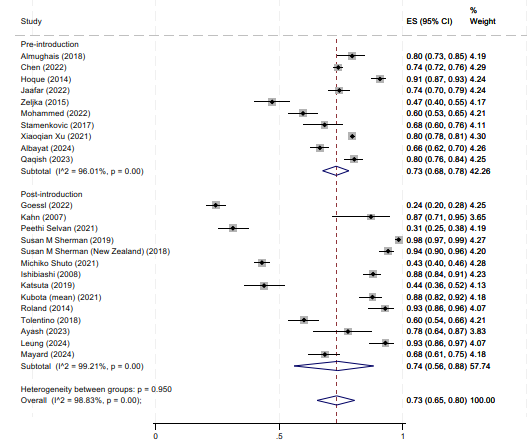


Proportion of healthcare workers recommendation willingness (intent) for both boys and girls by pre or post- official HPV vaccine introduction (n=24)


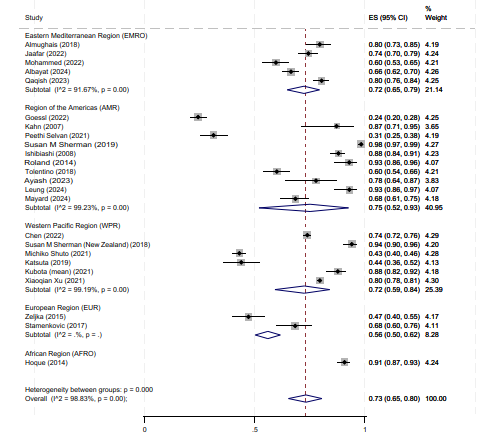


Proportion of healthcare workers recommendation willingness (intent) for both boys and girls by WHO region (n=24)


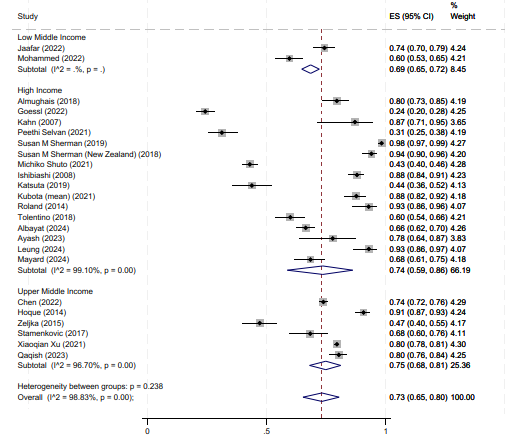


Proportion of healthcare workers recommendation willingness (intent) for both boys and girls by income level (n=24)
