# Supplementary material for: Healthcare worker practices for HPV vaccine recommendation: A systematic review and meta-analysis
Source: Hum Vaccin Immunother. 2024 Oct 14;20(1):2402122. doi: 10.1080/21645515.2024.2402122 (PMC11486212; doi:10.1080/21645515.2024.2402122)
Supplement: Appendix 5_Proportion of HCW recommendation behavior.docx [file KHVI_A_2402122_SM6229.docx]

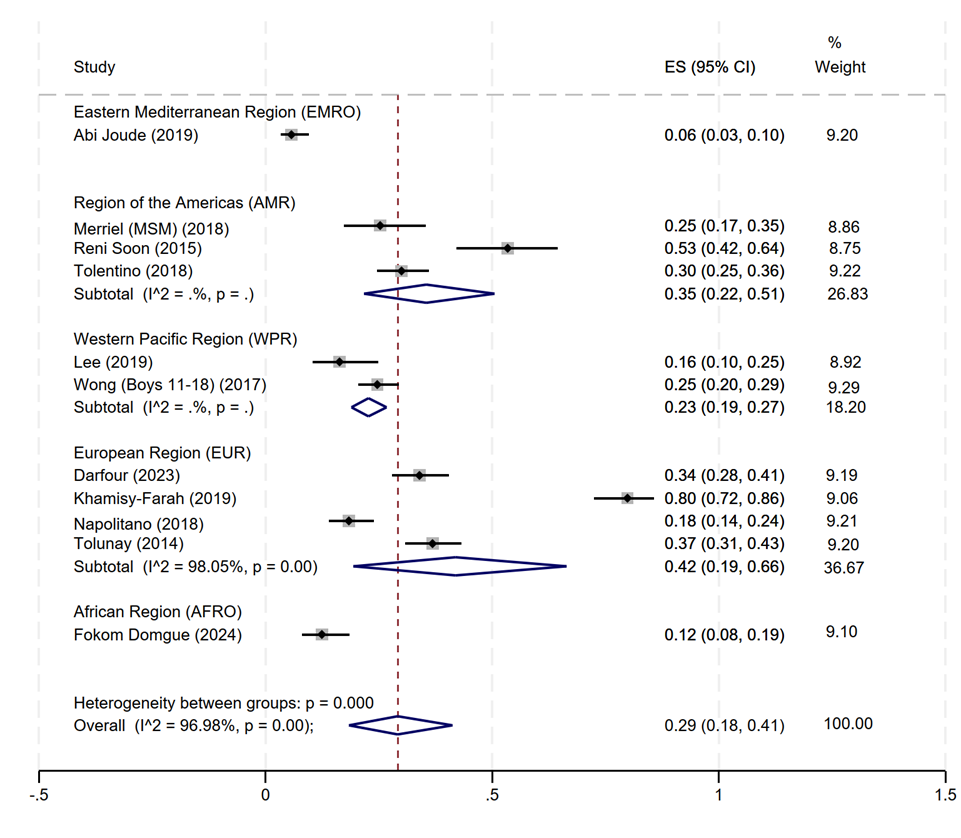


*Proportion of healthcare workers recommendation behaviour (practice) for both boys by WHO Region (n=11)*


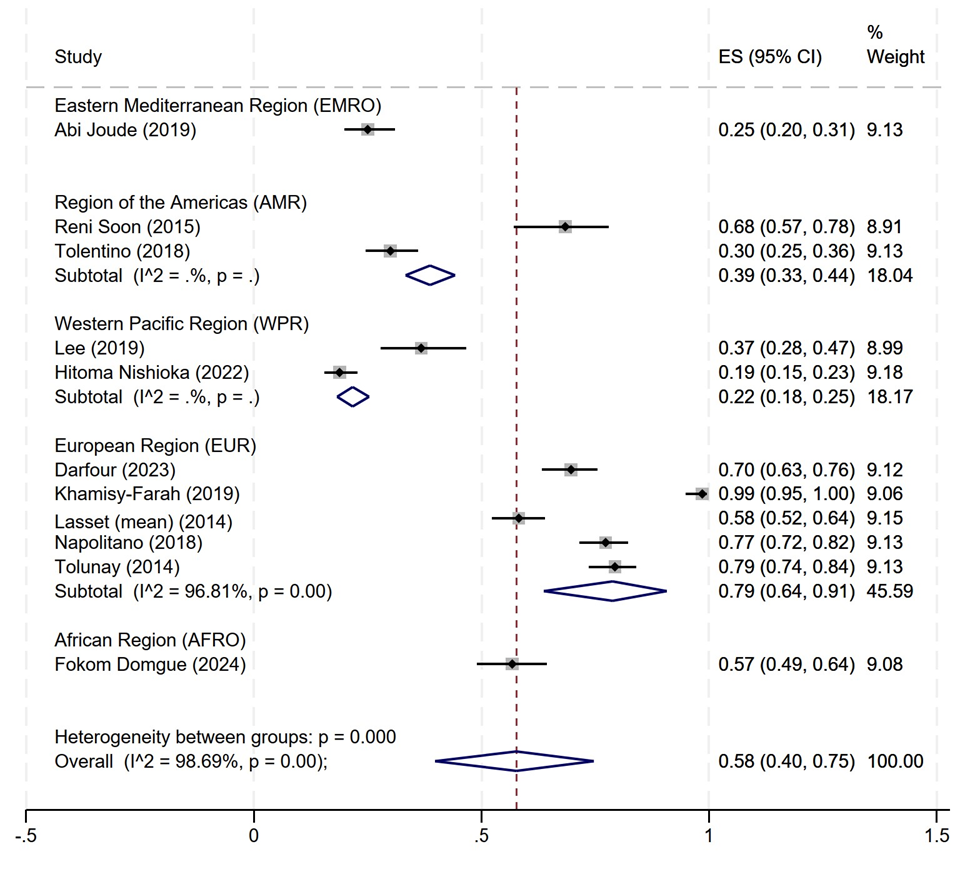
*Proportion of healthcare workers recommendation behaviour (practice) for girls by WHO Region (n=11)*


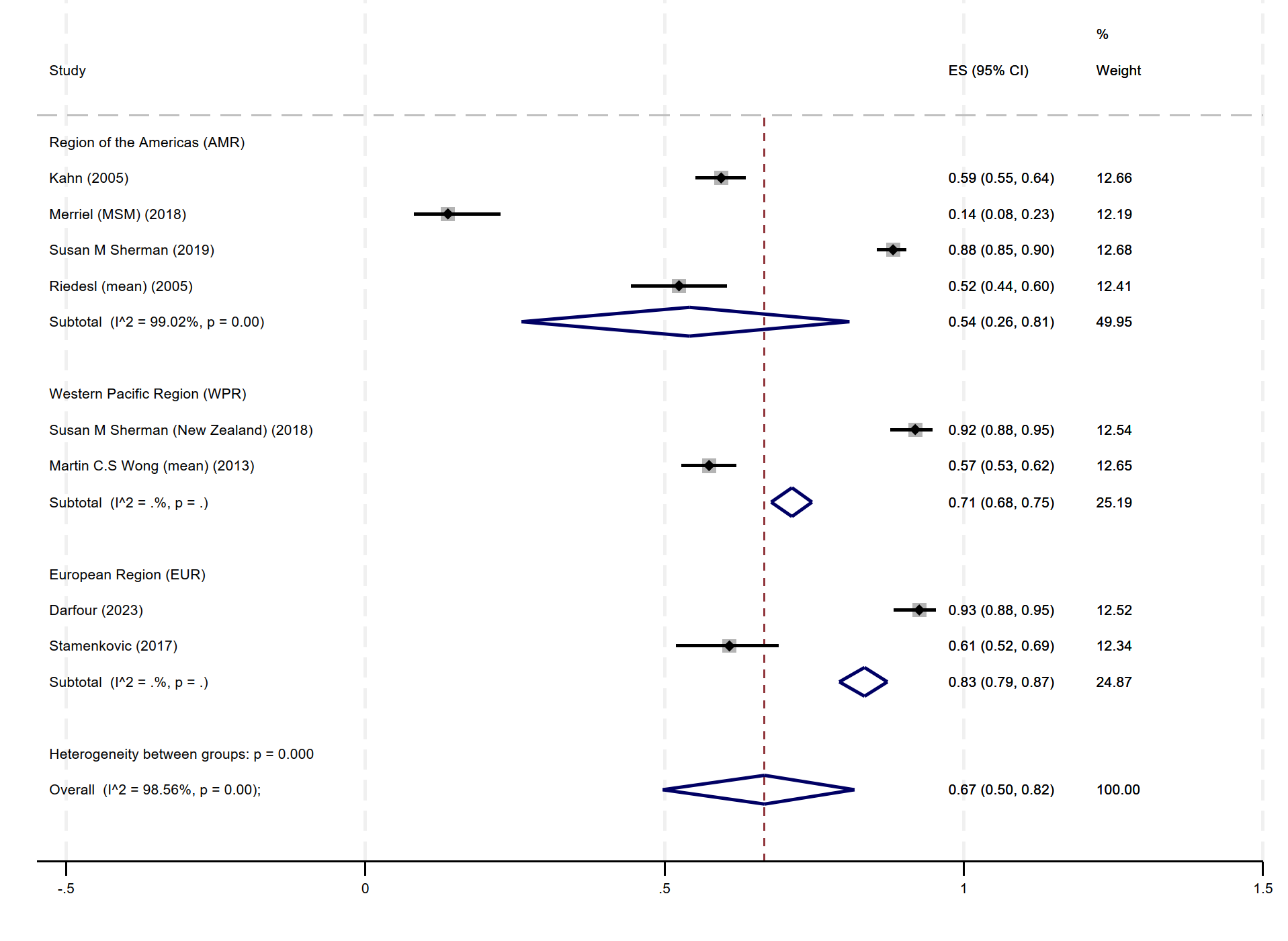
Proportion of healthcare workers recommendation willingness (intent) for boys by WHO region (n=4)


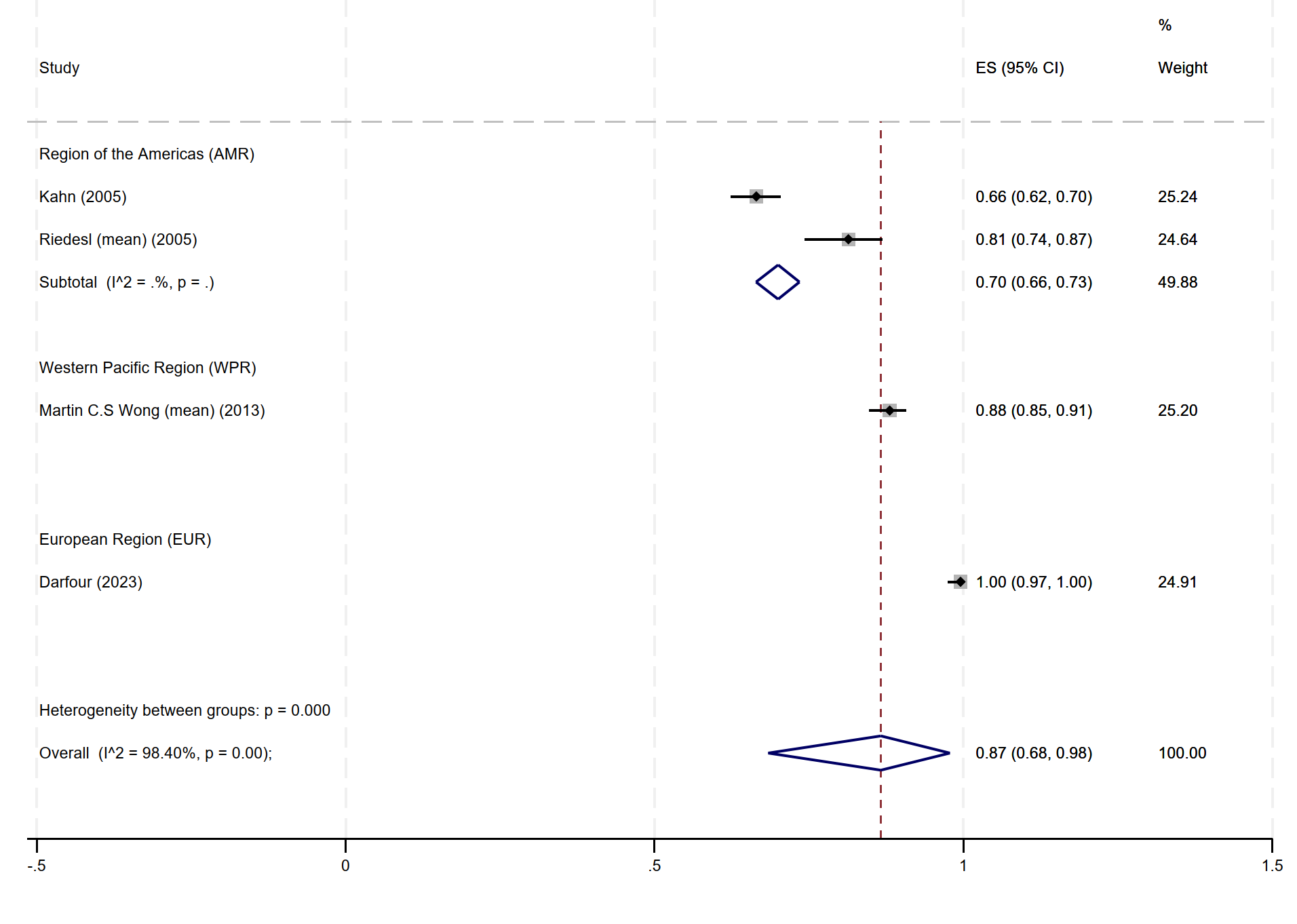
Proportion of healthcare workers recommendation willingness (intent) for girls by WHO region (n=4)
